# Cervical intraepithelial neoplasia and the risk of spontaneous preterm birth: A Dutch population-based cohort study with 45,259 pregnancy outcomes

**DOI:** 10.1371/journal.pmed.1003665

**Published:** 2021-06-04

**Authors:** Diede L. Loopik, Joris van Drongelen, Ruud L. M. Bekkers, Quirinus J. M. Voorham, Willem J. G. Melchers, Leon F. A. G. Massuger, Folkert J. van Kemenade, Albert G. Siebers

**Affiliations:** 1 Department of Obstetrics and Gynaecology, Radboud Institute for Molecular Life Sciences, Radboud university medical center, Nijmegen, the Netherlands; 2 Department of Obstetrics and Gynaecology, Radboud university medical center, Nijmegen, the Netherlands; 3 Department of Obstetrics and Gynaecology, Catharina Hospital, Eindhoven, the Netherlands; 4 PALGA, Houten, the Netherlands; 5 Department of Medical Microbiology, Radboud university medical center, Nijmegen, the Netherlands; 6 Department of Pathology, Erasmus Medical Center, Rotterdam, the Netherlands; 7 Department of Pathology, Radboud university medical center, Nijmegen, the Netherlands; The University of Edinburgh Usher Institute of Population Health Sciences and Informatics, UNITED KINGDOM

## Abstract

**Background:**

Excisional procedures of cervical intraepithelial neoplasia (CIN) may increase the risk of preterm birth. It is unknown whether this increased risk is due to the excision procedure itself, to the underlying CIN, or to secondary risk factors that are associated with both preterm birth and CIN. The aim of this study is to assess the risk of spontaneous preterm birth in women with treated and untreated CIN and examine possible associations by making a distinction between the excised volume of cervical tissue and having cervical disease.

**Methods and findings:**

This Dutch population-based observational cohort study identified women aged 29 to 41 years with CIN between 2005 and 2015 from the Dutch pathology registry (PALGA) and frequency matched them with a control group without any cervical abnormality based on age at and year of pathology outcome (i.e., CIN or normal cytology) and urbanization (<100,000 inhabitants or ≥100,000 inhabitants). All their 45,259 subsequent singleton pregnancies with a gestational age ≥16 weeks between 2010 and 2017 were identified from the Dutch perinatal database (Perined). Nineteen potential confounders for preterm birth were identified. Adjusted odds ratios (ORs) were calculated for preterm birth comparing the 3 different groups of women: (1) women without CIN diagnosis; (2) women with untreated CIN; and (3) women with treated CIN prior to each childbirth.

In total, 29,907, 5,940, and 9,412 pregnancies were included in the control, untreated CIN, and treated CIN group, respectively. The control group showed a 4.8% (1,002/20,969) proportion of spontaneous preterm birth, which increased to 6.9% (271/3,940) in the untreated CIN group, 9.5% (600/6,315) in the treated CIN group, and 15.6% (50/321) in the group with multiple treatments. Women with untreated CIN had a 1.38 times greater odds of preterm birth compared to women without CIN (95% confidence interval (CI) 1.19 to 1.60; *P* < 0.001). For women with treated CIN, these odds 2.07 times increased compared to the control group (95% CI 1.85 to 2.33; *P* < 0.001). Treated women had a 1.51 times increased odds of preterm birth compared to women with untreated CIN (95% CI 1.29 to 1.76; *P* < 0.001). Independent from cervical disease, a volume excised from the cervix of 0.5 to 0.9 cc increased the odds of preterm birth 2.20 times (37/379 versus 1,002/20,969; 95% CI 1.52 to 3.20; *P* < 0.001). These odds further increased 3.13 times and 5.93 times for women with an excised volume of 4 to 8.9 cc (90/724 versus 1,002/20,969; 95% CI 2.44 to 4.01; *P* < 0.001) and ≥9 cc (30/139 versus 1,002/20,969; 95% CI 3.86 to 9.13; *P* < 0.001), respectively. Limitations of the study include the retrospective nature, lack of sufficient information to calculate odds of preterm birth <24 weeks, and that the excised volume could only be calculated for a select group of women.

**Conclusions:**

In this study, we observed a strong correlation between preterm birth and a volume of ≥0.5 cc excised cervical tissue, regardless of the severity of CIN. Caution should be taken when performing excisional treatment in women of reproductive age as well as prudence in case of multiple biopsies. Fertile women with a history of performing multiple biopsies or excisional treatment for CIN may benefit from close surveillance during pregnancy.

## Abstract 2

In a population-based study, Diede Loopik and colleagues investigate associations between cervical intraepithelial neoplasia and risk of preterm birth among Dutch women.

## Author summary

**Why was this study done?:**

- Women who are treated for a precancerous cervical lesion may have an increased risk of preterm birth.
- It is unknown whether this increased risk is due to the treatment itself, to the cervical disease, or to secondary risk factors that are associated with both preterm birth and cervical disease.

**What did the researchers do and find?:**

- We identified women with untreated and treated cervical disease and matched them with a control group through the Dutch pathology registry and compared their pregnancy outcomes through the Dutch perinatal database.
- The control group (29,907 pregnancies) showed a 4.8% proportion of preterm birth, which increased to 6.9% in the untreated cervical disease group (5,940 pregnancies), 9.5% in the treated cervical disease group (9,412 pregnancies), and 15.6% in the group with multiple treatments (505 pregnancies).
- After adjustment for 19 potential confounders for preterm birth, women with untreated cervical disease had a 1.4 times and women with treated cervical disease had a more than 2 times increased odds of preterm birth compared to the control group.
- Independent from cervical disease, a volume excised from the cervix of 0.5 cc or more was associated with an approximately 2 times greater odds of preterm birth.

**What do these findings mean?:**

- We observed a strong association between women with a 0.5 cc or more excised cervical tissue volume (through biopsy and/or treatment) and the odds of preterm birth, regardless of severity of cervical disease or secondary risk factors associated with both preterm birth and cervical disease.
- Our findings suggest that caution should be taken when performing multiple biopsies or treatment for CIN in women of reproductive age and that women with such a history may benefit from close surveillance during pregnancy.

## Introduction

The introduction of cervical cancer screening programs has drastically decreased the incidence and mortality from cervical cancer, due to early treatment of high-grade cervical intraepithelial neoplasia (CIN) [1]. Women participating in the screening program may undergo biopsy or treatment within their reproductive age, as the incidence of CIN peaks at around the age of 30. Although women with a true risk of progression to cervical cancer (≥CIN3) need treatment, one should be conservative with excisional procedures if they have future pregnancy desires. Research has shown that excisional procedures of CIN may increase the risk of adverse pregnancy outcomes, such as preterm birth, preterm premature rupture of membranes (pPROM), and consequently less favorable neonatal outcomes as a direct result of prematurity [2–5]. Additionally, the number of excisional procedures and increasing cone depth further increase the risk of preterm birth [5].

Screening programs are replacing primary cytology testing by primary high-risk human papillomavirus (hrHPV) testing, resulting in higher referral rates and more women undergoing biopsy or treatment [6], which may further increase the number of women at risk for adverse pregnancy outcomes.

It is unknown whether the increased risk for preterm birth is due to the excision procedure itself, to the underlying CIN, or to secondary risk factors that are associated with both preterm birth and CIN. Literature shows conflicting results for the risk of preterm birth in treated women versus untreated women with CIN [3–5,7]. A recent meta-analysis including 69 publications concluded that many studies were of low quality; they are small, do not compare women in all 3 groups appropriately (women without a CIN diagnosis, with untreated CIN, and treated CIN), or have only limited information about potential confounders [5]. Additionally, as there are many reasons for premature induction of labor, it is important to identify women with spontaneous preterm birth.

A large study is needed with a comparison between women without CIN diagnosis, untreated CIN, and treated CIN, including adjustment for potential confounding factors. To examine the effect of diagnostic and therapeutic techniques, it is important to account for the volume excised from the cervix for each biopsy and treatment.

Therefore, the first aim of this population-based study is to assess the risk of spontaneous preterm birth in women with treated and untreated CIN compared to women without a CIN diagnosis and to examine the correlation of preterm birth with the extent of excised volume and having cervical disease, both adjusted for potential confounders. Secondly, the risk of other adverse maternal and neonatal outcomes is assessed.

## Methods

Women aged 29 to 41 years (including 3 screening invitations at age 30, 35, and 40) with a CIN diagnosis or adenocarcinoma in situ between 2005 and 2015 were identified from the Dutch pathology registry (PALGA) [8]. Only histologic outcomes of CIN obtained through biopsy or cervical excisional treatment were included. Cytological outcomes or histological outcomes diagnosed by hysterectomy, polypectomy, or endocervical curettage were excluded. The date and result of each biopsy and/or treatment was reported. The size of the excised cervical tissue was extracted from the macroscopic description when available using pattern recognition (length × width × depth) as recorded during laboratory processing. A maximum volume of 4.5 cc and 27 cc was accepted as plausible for biopsy and treatment, respectively. A frequency matched control group was identified from PALGA by selecting women with only normal cervical cytology and no CIN diagnosis in their history. Matching was done by age at and year of pathology outcome (i.e., CIN or normal cytology) and urbanization (<100,000 inhabitants or ≥100,000 inhabitants).

All singleton pregnancies with a gestational age ≥16 weeks between 2010 and 2017 were identified from the Dutch perinatal database (Perined). This database links all medical registries from the 4 professional organizations that provide perinatal care in the Netherlands: the Dutch Society of Obstetrics and Gynaecology (NVOG), the Royal Dutch Organisation of Midwives (KNOV), the Dutch Association of Pediatrics (NVK), and the National Association of General Practitioners (LHV). It contains detailed anonymized population-based information (over 97% of all pregnancies in the Netherlands) on pregnancies, deliveries, and neonatal (re)admissions [9].

The pseudonymized personal identifiers from PALGA and Perined were linked by Statistics Netherlands (CBS) using a dedicated Privacy Verzend Module (PVM), designed by as trusted third party (ZorgTTP). Unique women were given a new study number.

We used the gestational age of <37 weeks at birth for the primary outcome, spontaneous preterm birth. We compared the date of CIN diagnosis and date of birth to select only pregnancies occurring after CIN detection with or without prior treatment. We subanalyzed preterm birth for <28 and <32 weeks. Secondary (maternal) outcomes were spontaneous conception, threatened preterm birth, pPROM, assisted vaginal delivery, and cesarean section. Primary cesarean section and secondary cesarean section because of abnormal fetal position were excluded. Secondary (neonatal) outcomes were low birth weight (in grams and Hoftiezer percentiles), appearance, pulse, grimace, activity, and respiration (APGAR) score <7 at 5 minutes, high care or intensive care admission, and perinatal death (period defined as between 22 weeks of gestation and ≤28 days postpartum). Women with induction of labor were excluded for the outcomes of preterm birth, pPROM, and all neonatal outcomes. Ruptured membranes >36 hours before induction of labor were identified as spontaneous rupture of membranes and included in the analysis for pPROM. Risk factors for preterm birth were identified through an extensive literature search and expert consultation and were regarded as potential confounders. We identified age at childbirth, year of childbirth, ethnicity, diabetes mellitus, maternal infection, epilepsy, psychiatric diseases, history of abortion, history of preterm birth (excluding women with prior cervical excisional treatment), pregnancy by in vitro fertilization (IVF), nulliparous women, pre-eclampsia, gestational diabetes, placental abruption, placenta or vasa previa, congenital diseases, intrauterine growth restriction, macrosomia, stillbirth, and fetal distress as potential confounders for preterm birth. As these identified risk factors possibly could influence the outcome measures by unequal distribution among the 3 groups or may have an indirect association with having cervical disease, we chose to include all these potential confounders in the multivariable analysis. Urbanization was also included in multivariable analysis as the groups were composed differently when considering them per pregnancy instead of per woman.

Binary logistic regression was used to calculate odds ratios (ORs) with 95% confidence intervals (CIs). ORs were calculated for each outcome comparing the 3 different groups of women: (1) women without CIN; (2) women with untreated CIN; and (3) women with treated CIN. We also conducted a subanalysis for women with multiple excisional treatments. Women who gave birth multiple times could be included more than once in analysis. The total volume excised from the cervix (for example, the volume of the biopsy and following treatment or multiple biopsies) and severity of cervical disease was used to examine the possible correlations with preterm birth. We divided the volumes into quantiles and used trend analysis to find the optimal balance of variance and amount. Finally, we clustered the quantiles together into clinical relevant groups for the final regression. We considered different *P* values as statistically significant to adjust for multiple testing. This was done by dividing the 0.05 *P* value threshold by the number of tests done. All data preprocessing were performed through Python programming language in Jupyter Notebook with the use of “Pandas” version 1.0.1 and “NumPy” version 1.18.0 libraries (Python Software Foundation, version 3.7.4, https://www.python.org/) [10,11]. All statistical analyses were performed in the statistical software package SPSS 25.0 (IBM SPSS Statistics; New York, United States). The prospective analysis plan and changes in the plan can be found in the supporting information (S1 Protocol). This study is reported as per the Strengthening the Reporting of Observational Studies in Epidemiology (STROBE) guideline (S1 Checklist).

### Ethics approval

All the authors report adherence to ethical standards in the conception of the work, data collection, and writing of the manuscript. The study was approved by the scientific committee of the Dutch pathology registry (PALGA) and the Dutch perinatal registry (Perined). The study was exempt from institutional review board approval because data were gathered retrospectively and analyzed anonymously. As both registries and Statistics Netherlands (CBS) protect the anonymity of the included women and data were analyzed anonymously, their written consent was not needed.

### Patient and public involvement

Patients or the public were not involved in this study.

## Results

We identified 50,721 cytological and histological results in PALGA that had been linked to Perined. We excluded 321 misclassified outcomes and 112 histological outcomes obtained through hysterectomy, polypectomy, or endocervical curettage. The included 50,288 outcomes were from 39,036 women, which had 52,980 singleton pregnancies with a gestational age ≥16 weeks between 2010 and 2017. There were 7,721 pregnancies excluded, as they happened prior to the first CIN diagnosis. Eventually, 29,907 pregnancies were included in the control group, 5,940 pregnancies in the group of women with untreated CIN, and 9,412 pregnancies in the group of women with treated CIN (S1 Fig).

The basic characteristics of each group are described in Table 1. About half of the women in the untreated CIN group had CIN1, whereas more than 60% of the women had CIN3 in the treated CIN group. In 16% and 52% of the cases, the volume excised from the cervix before each childbirth could be determined in the untreated CIN and treated CIN group, respectively. The median volume for biopsy was 0.84 cc (range 0.10 to 4.44), and for excisional treatment 2.40 cc (range 0.10 to 26.30). The median gestation at spontaneous birth was 39+6 or 40+0 weeks (range 17+0 to 43+4). Women with untreated and treated CIN gave birth on average 2.85 days (95% CI 2.44 to 3.26; *P* < 0.001) and 1.13 days (95% CI 0.70 to 1.57; *P* < 0.001) earlier compared to women without CIN. Women with ≥2 times treated CIN gave birth on average 7.03 days (95% CI 4.81 to 9.25; *P* <0.001) earlier compared to women without CIN.

**Table 1:** Basic characteristics of women with no CIN, untreated CIN, and treated CIN before each childbirth.

	No CIN *n* = 29,907	Untreated CIN *n =* 5,940	Treated CIN *n* = 9,412
**Age of women at birth**, years			
Median (range)	33.0 (24–48)	34.0 (29–46)	34.0 (29–49)
**Year of childbirth**			
Range	2010–2017	2010–2017	2010–2017
**Urbanization**, n (%)			
<100,000 inhabitants	18,095 (60.5)	3,076 (51.8)	5,083 (54.0)
≥100,000 inhabitants	11,812 (39.5)	2,864 (48.2)	4,329 (46.0)
**Number of treatments**, n (%)			
1	NA	NA	8,907 (94.6)
2	NA	NA	461 (4.9)
≥3	NA	NA	44 (0.5)
**Highest diagnosis**, n (%)			
CIN1	NA	3,037 (51.1)	651 (6.9)
CIN2	NA	1,593 (26.8)	2,684 (28.5)
CIN3	NA	1,268 (21.4)^a^	5,865 (62.3)
AIS	NA	31 (0.5)^a^	145 (1.5)
Cervical cancer	NA	11 (0.2)^a^	67 (0.7)
**Volume taken from cervix**			
n (%)	NA	971 (16.3)	4,865 (51.7)
Mean (SD), cc	NA	1.16 (1.06)	3.13 (2.78)
Median (range), cc	NA	0.84 (0.10–4.44)	2.40 (0.10–26.30)
**Gravidity**			
Median, (range)	2 (1–14)	2 (1–17)	2 (1–20)
**Parity**			
Median (range)	1 (0–14)	1 (0–16)	1 (0–18)
**Gestational age at birth**^b^			
n (%)	20,969 (70.1)	3,940 (66.3)	6,315 (67.1)
Median (range), weeks	40+0 (20+0 to 42+5)	39+6 (17+6 to 43+4)	39+6 (17+0 to 42+3)

^a^Patients could have been lost to follow-up or have received other forms of treatment without histologic results, such as radiotherapy, cryotherapy, electrocoagulation, laser ablation, or a topical immune-response modulator.

^b^Women with induction of labor were excluded from analysis.

Abbreviations: AIS, adenocarcinoma in situ; CIN, cervical intraepithelial neoplasia; NA, not applicable; SD, standard deviation.

### Preterm birth

**Fig 1:**
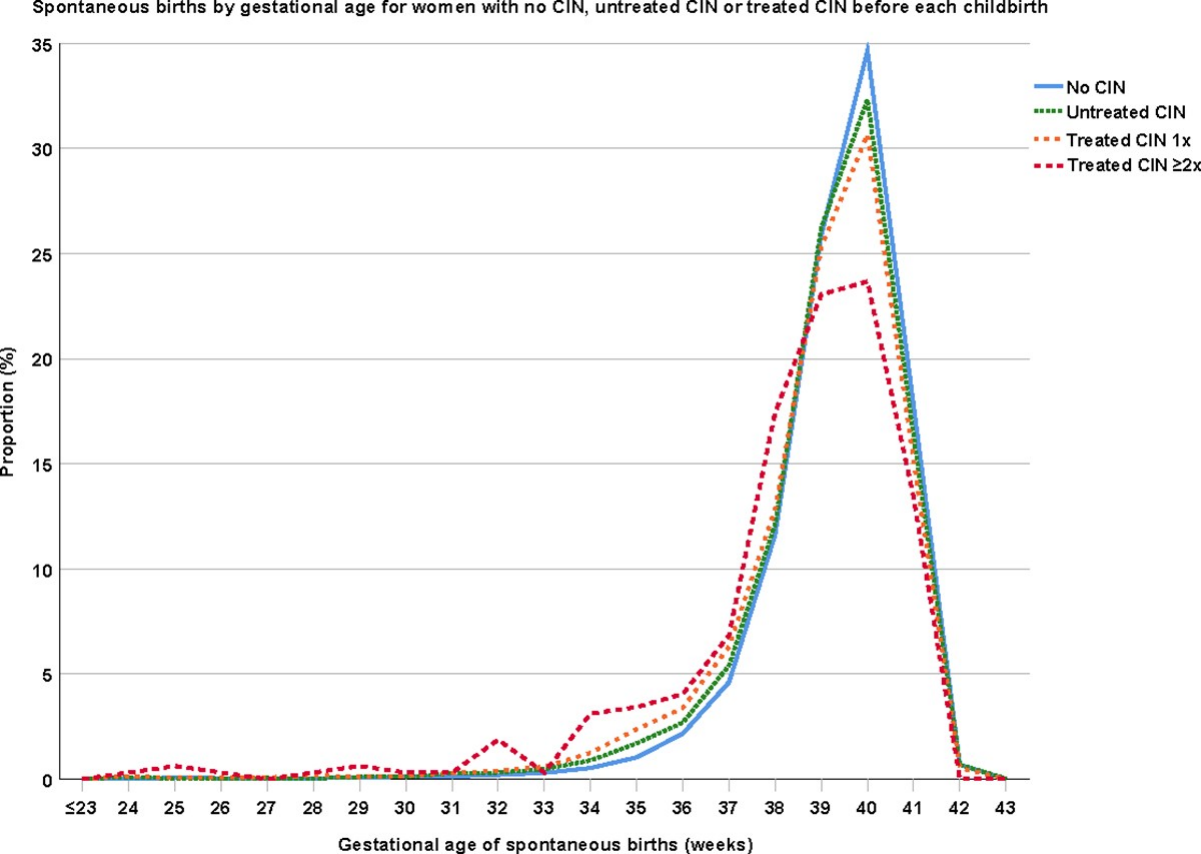
Spontaneous births by gestational age for women with no CIN, untreated CIN, or treated CIN (treated CIN 1× or treated CIN ≥2×) before each childbirth. The distribution between all 4 groups was statistically significantly different (all *P* < 0.001; two-sample Kolmogorov–Smirnov test). CIN, cervical intraepithelial neoplasia.

Fig 1 shows the proportion of spontaneous births in women without CIN, with untreated CIN, single treated CIN, and multiple treated CIN per gestational age. The risk of preterm birth between these groups of women is shown in Table 2 (unadjusted analyses in S2 Table). Overall, 1,873 (6.0%), 228 (0.7%), and 85 (0.3%) women delivered their baby before 37, 32, and 28 weeks, respectively. The control group showed a 4.8% proportion of preterm birth <37 weeks, which increased to 6.9% in the untreated CIN group, 9.5% in the treated CIN group, and 15.6% in the group with multiple treatments. After adjustment for all potential confounders (age at childbirth, year of childbirth, urbanization, ethnicity, diabetes mellitus, maternal infection, epilepsy, psychiatric diseases, history of abortion, history of preterm birth, pregnancy by IVF, nulliparous women, pre-eclampsia, gestational diabetes, placental abruption, placenta or vasa previa, congenital diseases, intrauterine growth restriction, macrosomia, stillbirth, and fetal distress), women with untreated CIN had a 1.38 times increased odds of preterm birth compared to women without CIN (95% CI 1.19 to 1.60; *P* < 0.001). For women with treated CIN, these odds were 2.07 times greater compared to the control group (95% CI 1.85 to 2.33; *P* < 0.001). Treated women had a 1.51 times higher odds of preterm birth compared to women with untreated CIN (95% CI 1.29 to 1.76; *P* < 0.001). The odds further increased for women with multiple treatments (compared to the control group: OR 3.66; 95% CI 2.66 to 5.05; *P* < 0.001; compared to untreated CIN: OR 2.66; 95% CI 1.90 to 3.72; *P* < 0.001; and compared to women with single treatment: OR 1.83; 95% CI 1.33 to 2.53; *P* < 0.001). No statistically significant difference was found for preterm birth <32 and <28 weeks for women with untreated CIN compared to women without CIN or treated CIN (<32 weeks untreated CIN versus no CIN: OR 1.34; 95% CI 0.88 to 2.05; *P* = 0.18; <32 weeks treated CIN versus untreated CIN: OR 1.72; 95% CI 1.12 to 2.65; *P* = 0.01; <28 weeks untreated CIN versus no CIN: OR 1.22; 95% CI 0.58 to 2.57; *P* = 0.60; and <28 weeks treated CIN versus untreated CIN: OR 2.09; 95% CI 0.99 to 4.40; *P* = 0.015). The odds of preterm birth <32 and <28 weeks remained statistically significantly increased for women with treated CIN compared to the control group (OR 2.30; 95% CI 1.68 to 3.16; *P* < 0.001 and OR 2.55; 95% CI 1.50 to 4.35; *P* = 0.001, respectively). The OR of preterm birth <32 and <28 weeks was the highest for women with multiple treatments compared to women without CIN (OR 5.32; 95% CI 2.58 to 10.95; *P* < 0.001 and OR 7.02; 95% CI 2.35 to 21.02; *P* < 0.001, respectively). The univariate logistic regression analyses for preterm birth for the 19 potential confounders can be found in S1 Table.

**Table 2:** Multivariable logistic regression for preterm birth for women with no CIN, untreated CIN, and treated CIN before each childbirth^a^.

Primary outcome	Event/total (%)		OR (95% CI)	*P* value^c^
Preterm birth <37 weeks^b^	1,873/31,224 (6.0)			
Untreated CIN vs no CIN	271/3,940 (6.9)	1,002/20,969 (4.8)	1.38 (1.19 to 1.60)	<0.001*
Treated CIN vs no CIN	600/6,315 (9.5)	1,002/20,969 (4.8)	2.07 (1.85 to 2.33)	<0.001*
Treated CIN vs untreated CIN	600/6,315 (9.5)	271/3,940 (6.9)	1.51 (1.29 to 1.76)	<0.001*
Treated CIN ≥2× vs no CIN	50/321 (15.6)	1,002/20,969 (4.8)	3.66 (2.66 to 5.05)	<0.001*
Treated CIN ≥2× vs untreated CIN	50/321 (15.6)	271/3,940 (6.9)	2.66 (1.90 to 3.72)	<0.001*
Treated CIN ≥2× vs treated CIN 1×	50/321 (15.6)	550/5,994 (9.2)	1.83 (1.33 to 2.53)	<0.001*
**Preterm birth <32 weeks**^b^	228/31,224 (0.7)			
Untreated CIN vs no CIN	32/3,940 (0.8)	116/20,969 (0.6)	1.34 (0.88 to 2.05)	0.18
Treated CIN vs no CIN	80/6,315 (1.3)	116/20,969 (0.6)	2.30 (1.68 to 3.16)	<0.001*
Treated CIN vs untreated CIN	80/6,315 (1.3)	32/3,940 (0.8)	1.72 (1.12 to 2.65)	0.01
Treated CIN ≥2× vs no CIN	9/321 (2.8)	116/20,969 (0.6)	5.32 (2.58 to 10.95)	<0.001*
Treated CIN ≥2× vs untreated CIN	9/321 (2.8)	32/3,940 (0.8)	3.95 (1.82 to 8.61)	<0.001*
Treated CIN ≥2× vs treated CIN 1×	9/321 (2.8)	71/5,994 (1.2)	2.47 (1.19 to 5.12)	0.02
**Preterm birth <28 weeks**^b^	85/31,224 (0.3)			
Untreated CIN vs no CIN	12/3,940 (0.3)	41/20,969 (0.2)	1.22 (0.58 to 2.57)	0.60
Treated CIN vs no CIN	32/6,315 (0.5)	41/20,969 (0.2)	2.55 (1.50 to 4.35)	0.001*
Treated CIN vs untreated CIN	32/6,315 (0.5)	12/3,940 (0.3)	2.09 (0.99 to 4.40)	0.05
Treated CIN ≥2× vs no CIN	<5/321 (<1.6)^d^	41/20,969 (0.2)	7.02 (2.35 to 21.02)	<0.001*
Treated CIN ≥2× vs untreated CIN	<5/321 (<1.6)^d^	12/3,940 (0.3)	5.72 (1.71 to 19.16)	0.005*
Treated CIN ≥2× vs treated CIN 1×	<5/321 (<1.6)^d^	28/5,994 (0.5)	3.03 (1.01 to 9.09)	0.05

^a^With adjustment for age at childbirth, year of childbirth, urbanization, ethnicity, diabetes mellitus, maternal infection, epilepsy, psychiatric diseases, history of abortion, history of preterm birth, pregnancy by IVF, nulliparous women, pre-eclampsia, gestational diabetes, placental abruption, placenta or vasa previa, congenital diseases, intrauterine growth restriction, macrosomia, stillbirth, and fetal distress.

^b^Women with induction of labor were excluded from analysis.

^c^To adjust for multiple testing, we considered a *P* value of <0.008 statistically significant.

^d^To prevent revealing data, numbers of less than 5 are grouped together, conform the rules of CBS.

*Statistically significant.

Abbreviations: CBS, Statistics Netherlands; CI, confidence interval; CIN, cervical intraepithelial neoplasia; IVF, in vitro fertilization; NA, not applicable; OR, odds ratio.

### Examining excision of cervical tissue and having cervical disease as possible associations of preterm birth

**Fig 2:**
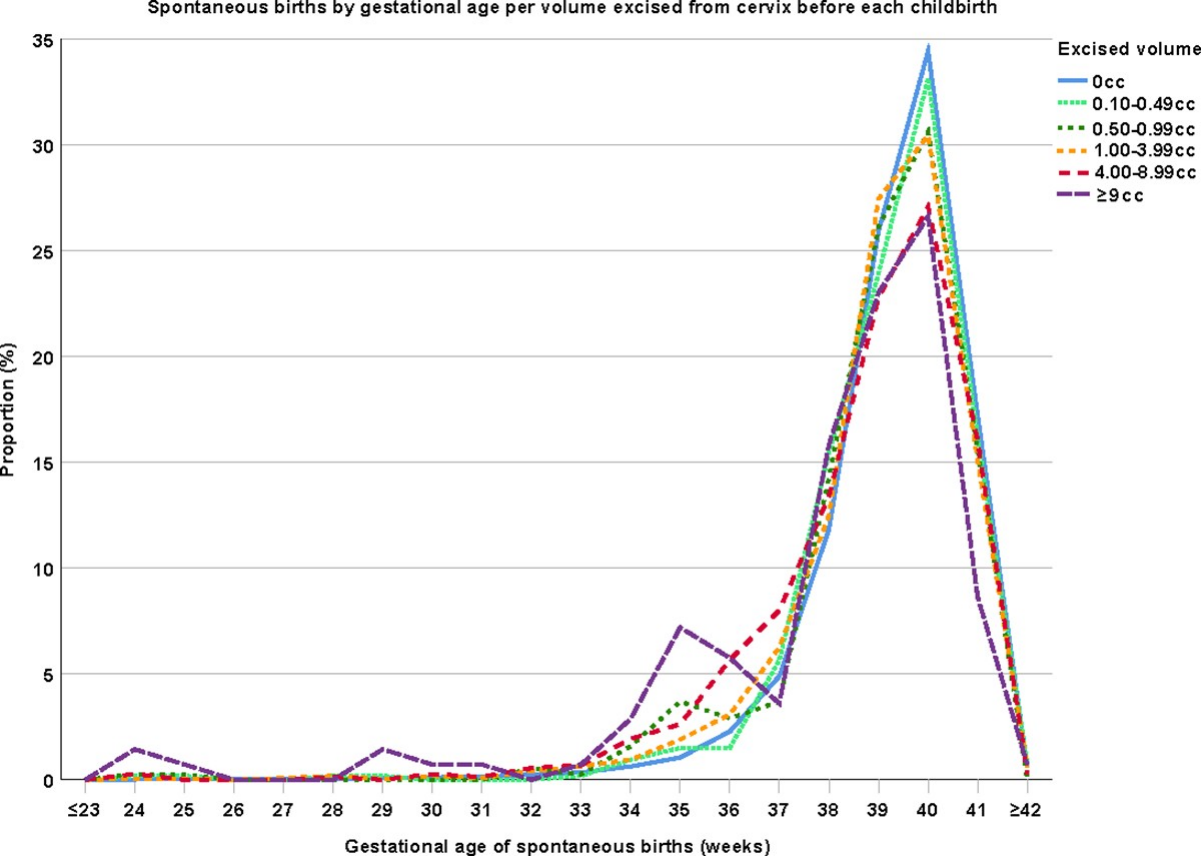
Spontaneous births by gestational age per volume excised from cervix before each childbirth. The distribution between the groups was statistically significantly different for 0 cc vs 1.00–3.99 cc (*P* < 0.001), 0 cc vs 4.00–8.99 cc (*P* < 0.001), 0 cc vs ≥9 cc (*P* < 0.001), 0.10–0.49 cc vs 4.00–8.99 cc (*P* = 0.005), 0.10–0.49 cc vs ≥9 cc (*P* = 0.004), 1.00–3.99 cc vs 4.00–8.99 cc (*P* = 0.005), 1.00–3.99 cc vs ≥9 cc (*P* = 0.008) (two-sample Kolmogorov–Smirnov test). CIN, cervical intraepithelial neoplasia.

Fig 2 shows the proportion of spontaneous births per gestational age stratified per volume excised from the cervix. To distinguish if the increased odds for preterm birth in treated versus untreated women with CIN are associated with the volume of the excised tissue or the severity of cervical disease, we performed a multivariable logistic regression including these variables. The odds of preterm birth remained significant with adjustment for severity of cervical disease (OR 1.40; 95% CI 1.17 to 1.67; *P* < 0.001) but were no longer statistically significant after adjustment for size of the excision (OR 1.22 95% CI 0.87 to 1.73; *P* = 0.25). Per 1 cc of excised volume of the cervix women gave birth on average a half day earlier (Control group included: 0.54 day; 95% CI 0.30 to 0.77; *P* < 0.001. Control group excluded: 0.52 day; 95% CI 0.28 to 0.76; *P* < 0.001). In Table 3, we assessed if there was a cutoff value for volume from which the risk shows a significant increase (unadjusted analyses in S3 Table). Independent from the severity of cervical disease, an excised volume between 0.10 and 0.49 cc did not increase the odds of preterm birth (OR 1.06; 95% CI 0.69 to 1.64; *P* = 0.79). However, a ±2 times increased odds of preterm birth was seen from a volume of 0.50 cc (an excised volume of 0.50 to 0.99 cc: OR 2.20; 95% CI 1.52 to 3.20; *P* < 0.001; and an excised volume of 1.00 to 3.99 cc: OR 1.70; 95% CI 1.40 to 2.08; *P* < 0.001). These odds further increased for women with an excised volume of 4.00 to 8.99 cc (OR 3.13; 95% CI 2.44 to 4.01; *P* < 0.001) and ≥9.00 cc (OR 5.93; 95% CI 3.86 to 9.13; *P* < 0.001). The other way around, we did not find a statistically significant difference in the odds of preterm birth in women with CIN1 or CIN2 compared to women without CIN (OR 1.13; 95% CI 0.75 to 1.71; *P* = 0.57 and OR 1.30; 95% CI 1.02 to 1.67; *P* = 0.04, respectively), or between women with CIN2 or ≥CIN3 compared to CIN1 (OR 1.11; 95% CI 0.70 to 1.74; *P* = 0.66 and OR 1.21; 95% CI 0.79 to 1.85; *P* = 0.39, respectively), when we adjusted for the volume taken from the cervix (S4 Table). Only women with ≥CIN3 had a greater odds of preterm birth compared to women without CIN independently from the volume excised from the cervix (OR 1.44; 95% CI 1.17 to 1.77; *P* < 0.001).

**Table 3:** Multivariable logistic regression for preterm birth per volume taken from the cervix before each childbirth^a^.

Preterm birth <37 weeks^b^	Events/total (%)		OR (95% CI)	*P* value^c^
	1,355/24,950 (5.4)			
0.10–0.49 cc vs 0 cc	26/531 (4.9)	1,002/20,969 (4.8)	1.06 (0.69 to 1.64)	0.79
0.50–0.99 cc vs 0 cc	37/379 (9.8)	1,002/20,969 (4.8)	2.20 (1.52 to 3.20)	<0.001*
1.00–3.99 cc vs 0 cc	170/2,208 (7.7)	1,002/20,969 (4.8)	1.70 (1.40 to 2.08)	<0.001*
4.00–8.99 cc vs 0 cc	90/724 (12.4)	1,002/20,969 (4.8)	3.13 (2.44 to 4.01)	<0.001*
≥9 cc vs 0 cc	30/139 (21.6)	1,002/20,969 (4.8)	5.93 (3.86 to 9.13)	<0.001*

^a^With adjustment for age at childbirth, year of childbirth, urbanization, severity of cervical disease (normal, CIN1, CIN2, or ≥CIN3), ethnicity, diabetes mellitus, maternal infection, epilepsy, psychiatric diseases, history of abortion, history of preterm birth, pregnancy by IVF, nulliparous women, pre-eclampsia, gestational diabetes, placental abruption, placenta or vasa previa, congenital diseases, intrauterine growth restriction, macrosomia, stillbirth, and fetal distress.

^b^Women with induction of labor were excluded from analysis.

^c^To adjust for multiple testing, we considered a *P* value of <0.01 statistically significant.

*Statistically significant.

Abbreviations: CI, confidence interval; CIN, cervical intraepithelial neoplasia; IVF, in vitro fertilization; OR, odds ratio.

### Maternal outcomes

In Table 4 and S5 Table, we show the maternal outcomes with the same group comparisons as for our primary outcome. As expected, we found similar ORs for threatened preterm birth and pPROM as we saw for preterm birth. There was no significant difference in odds for spontaneous conception between women with treated CIN compared to untreated CIN (OR 0.94; 95% CI 0.81 to 1.09; *P* = 0.43). We found no statistically significant differences in the odds of assisted vaginal delivery or cesarean section between all groups, beside a slightly higher odds for assisted vaginal delivery in treated women compared to women without CIN (OR 1.18; 95% CI 1.08 to 1.29; *P* < 0.001).

**Table 4:** Multivariable logistic regression for maternal and neonatal outcomes for women with no CIN, untreated CIN, and treated CIN before each childbirth^a^.

**Maternal outcomes**				
	**Event/total (%)**		**OR (95% CI)**	***P* value**^**c**^
**Spontaneous conception**^b^	27,938/30,087 (92.9)			
Untreated CIN vs no CIN	3,710/4,017 (92.4)	18,336/19,668 (93.2)	1.27 (1.11 to 1.45)	0.001*
Treated CIN vs no CIN	5,892/6,402 (92.0)	18,336/19,668 (93.2)	1.19 (1.07 to 1.33)	0.002*
Treated CIN vs untreated CIN	5,892/6,402 (92.0)	3,710/4,017 (92.4)	0.94 (0.81 to 1.09)	0.43
**Threatened preterm birth**	1,247/45,259 (2.8)			
Untreated CIN vs no CIN	192/5,940 (3.2)	659/29,907 (2.2)	1.53 (1.29 to 1.81)	<0.001*
Treated CIN vs no CIN	396/9,412 (4.2)	659/29,907 (2.2)	2.08 (1.81 to 2.38)	<0.001*
Treated CIN vs untreated CIN	396/9,412 (4.2)	192/5,940 (3.2)	1.36 (1.14 to 1.62)	0.001*
**pPROM**^d^	1,971/30,740 (6.4)			
Untreated CIN vs no CIN	292/3,953 (7.4)	1,045/20,386 (5.1)	1.31 (1.13 to 1.51)	<0.001*
Treated CIN vs no CIN	634/6,401 (9.9)	1,045/20,386 (5.1)	1.90 (1.70 to 2.12)	<0.001*
Treated CIN vs untreated CIN	634/6,401 (9.9)	292/3,953 (7.4)	1.45 (1.25 to 1.67)	<0.001*
**Assisted vaginal delivery**	3,941/44,556 (8.8)			
Untreated CIN vs no CIN	556/5,832 (9.5)	2,453/29,482 (8.3)	1.04 (0.94 to 1.16)	0.42
Treated CIN vs no CIN	932/9,242 (10.1)	2,453/29,482 (8.3)	1.18 (1.08 to 1.29)	<0.001*
Treated CIN vs untreated CIN	932/9,242 (10.1)	556/5,832 (9.5)	1.13 (1.01 to 1.27)	0.04
**Cesarean section**^e^	3,640/44,157 (8.2)			
Untreated CIN vs no CIN	518/5,777 (9.0)	2,343/29,231 (8.0)	0.94 (0.85 to 1.04)	0.23
Treated CIN vs no CIN	779/9,149 (8.5)	2,343/29,231 (8.0)	0.91 (0.83 to 0.99)	0.03
Treated CIN vs untreated CIN	779/9,149 (8.5)	518/5,777 (9.0)	0.97 (0.86 to 1.09)	0.55
**Neonatal outcomes**				
	**Event/total (%)**		**OR (95% CI)**	***P* value**^**c**^
**Birth weight <2,500 grams**^f^	1,126/31,186 (3.6)			
Untreated CIN vs no CIN	171/3,936 (4.3)	573/20,942 (2.7)	1.45 (1.20 to 1.76)	<0.001*
Treated CIN vs no CIN	382/6,308 (6.1)	573/20,942 (2.7)	2.21 (1.91 to 2.56)	<0.001*
Treated CIN vs untreated CIN	382/6,308 (6.1)	171/3,936 (4.3)	1.52 (1.25 to 1.85)	<0.001*
**Birth weight <1,500 grams**^f^	153/31,186 (0.5)			
Untreated CIN vs no CIN	20/3,936 (0.5)	77/20,942 (0.4)	1.14 (0.66 to 1.95)	0.65
Treated CIN vs no CIN	56/6,308 (0.9)	77/20,942 (0.4)	2.32 (1.58 to 3.41)	<0.001*
Treated CIN vs untreated CIN	56/6,308 (0.9)	20/3,936 (0.5)	2.04 (1.18 to 3.54)	0.01
**Birth weight Hoftiezer percentile ≤2**^f^	542/31,084 (1.7)			
Untreated CIN vs no CIN	87/3,917 (2.2)	334/20,877 (1.6)	0.87 (0.66 to 1.13)	0.28
Treated CIN vs no CIN	121/6,290 (1.9)	334/20,877 (1.6)	1.03 (0.81 to 1.30)	0.83
Treated CIN vs untreated CIN	121/6,290 (1.9)	87/3,917 (2.2)	1.19 (0.88 to 1.60)	0.27
**APGAR <7**^f^	400/31,224 (1.3)			
Untreated CIN vs no CIN	57/3,940 (1.4)	232/20,969 (1.1)	1.18 (0.87 to 1.61)	0.29
Treated CIN vs no CIN	111/6,315 (1.8)	232/20,969 (1.1)	1.47 (1.15 to 1.88)	0.002*
Treated CIN vs untreated CIN	111/6,315 (1.8)	57/3,940 (1.4)	1.24 (0.89 to 1.73)	0.20
**NICU admission**^f^	827/31,224 (2.6)			
Untreated CIN vs no CIN	104/3,940 (2.6)	480/20,969 (2.3)	1.03 (0.82 to 1.29)	0.79
Treated CIN vs no CIN	243/6,315 (3.8)	480/20,969 (2.3)	1.56 (1.32 to 1.85)	<0.001*
Treated CIN vs untreated CIN	243/6,315 (3.8)	104/3,940 (2.6)	1.52 (1.19 to 1.93)	0.001*
**Perinatal death**^f^^,^^g^	56/31,216 (0.2)			
Untreated CIN vs no CIN	<5/3,937 (<0.2)^h^	41/20,967 (0.2)	0.37 (0.11 to 1.20)	0.10
Treated CIN vs no CIN	11/6,312 (0.2)	41/20,967 (0.2)	0.64 (0.30 to 1.38)	0.25
Treated CIN vs untreated CIN	11/6,312 (0.2)	<5/3,937 (<0.2)^h^	1.74 (0.48 to 6.38)	0.40

^a^With adjustment for age at childbirth, year of childbirth, urbanization, ethnicity, diabetes mellitus, maternal infection, epilepsy, psychiatric diseases, history of abortion, history of preterm birth, pregnancy by IVF, nulliparous women, pre-eclampsia, gestational diabetes, placental abruption, placenta or vasa previa, congenital diseases, intrauterine growth restriction, macrosomia, stillbirth, and fetal distress.

^b^Adjustment for pregnancy by IVF excluded.

^c^To adjust for multiple testing, we considered a *P* value of <0.008 statistically significant.

^d^Women with induction of labor with ≤36 hours of rupture of membranes were excluded from analysis.

^e^Women with a primary cesarean section and women with the position of the baby being the indication for secondary cesarean section were excluded.

^f^Women with induction of labor were excluded from analysis.

^g^Period defined as between 22 weeks of gestation and ≤28 days postpartum.

^h^To prevent revealing data, numbers of less than 5 are grouped together, conform the rules of CBS.

*Statistically significant.

Abbreviations: APGAR, appearance, pulse, grimace, activity, and respiration; CBS, Statistics Netherlands; CI, confidence interval; CIN, cervical intraepithelial neoplasia; IVF, in vitro fertilization; NICU, neonatal intensive care unit; pPROM, preterm premature rupture of membranes.

### Neonatal outcomes

Neonatal outcomes are shown in Table 4 and S6 Table. Given the positive correlation between gestational age and birth weight, similar ORs were found for low birth weight (<2,500 grams) as for preterm birth. No statistically significant difference was found for birth weight <2,000 grams for women with untreated CIN compared to women without CIN (OR 1.50; 95% CI 1.08 to 2.08; *P* = 0.02) or treated CIN compared to untreated CIN (OR 1.54; 95% CI 1.10 to 2.16; *P* = 0.01). The highest odds for birth weight <1,000 grams was for women with multiple treatments compared to women without CIN (OR 8.30; 95% CI 2.74 to 25.19; *P* < 0.001). The Hoftiezer percentiles show that there was no statistically significant difference in the odds of small for gestational age. A low APGAR score and intensive care admission rates were all increased in women with treated CIN compared to women without CIN (OR 1.47; 95% CI 1.15 to 1.88; *P* = 0.002 and OR 1.56;95% CI 1.32 to 1.85; *P* < 0.001, respectively). Perinatal death was too rare to give conclusive results (overall 56/31,216; 0.2%).

## Discussion

### Main outcome

In this population-based study, including 45,259 pregnancy outcomes, we observe that both women with untreated CIN and treated CIN have an increased odds of spontaneous preterm birth compared to women without a CIN diagnosis. We also observe that an excised total volume of <0.5 cc of cervical tissue is not associated with preterm birth; however, a higher excised volume is linked to a ≥2 times increase in odds of preterm birth, which seems independent from the severity of the underlying cervical disease or other potential risk factors.

### Treated women versus women without CIN

In our study, we observed that in women with treated CIN, the odds of preterm birth <37 weeks doubles as compared to healthy controls. The most recent Cochrane review showed a comparable risk of preterm birth in treated women compared to an untreated external comparison group (risk ratio (RR) 1.97 [95% CI 1.71 to 2.26]) [5]. For preterm birth <32 and <28 weeks, they only compared excisional treatment to all women without treatment (RR 2.48 [95% CI 1.92 to 3.20] and RR 2.81 [95% CI 1.89 to 4.18], respectively), but results are overall comparable with our findings. Only 2 large studies [12,13] and several smaller studies [14–25] assessed the risk of spontaneous preterm birth, excluding women with premature induction of labor, which is the actual outcome of interest. The same meta-analysis found a RR of 1.76 (95% CI 1.47 to 2.11) for spontaneous preterm birth in treated women versus women without treatment [5]. Only 7 and 2 studies were assessed for preterm birth <32 and <28 weeks, respectively, with increasing RRs in more severe preterm birth [5].

### Treated women versus untreated women with CIN

Over the years, it has become more clear that not only women with excisional treatment of CIN but also women with conservatively managed CIN may have an increased risk of adverse perinatal outcomes. We observed an increased adjusted odds for preterm birth in treated women versus untreated women with CIN. These increased odds remained statistically significant with adjustment for severity of cervical disease but were no longer statistically significant after adjustment for size of the excision. This is in line with the Cochrane review that found an increased risk in women with untreated CIN compared to the general population [5]. However, 3 other meta-analyses found an increased, although not significant, risk of preterm birth for women with treated CIN versus untreated CIN [3–5,7]. These findings raise the question whether having cervical disease or underlying common risk factors are actually causing preterm birth. While some studies have found no association between treated CIN and preterm birth after adjustment of potential confounders [23,25–27], our results remained significant after adjustment for 19 potential confounding factors. These results suggest that the possible correlation of preterm birth points to the excisional procedure itself (biopsy or treatment) and not the underlying cervical disease.

### Size of the excision

It seems logical that the risk of preterm birth depends on the excised cervical volume. We observed that the turning point of an excision without increasing the odds of preterm birth could be from 0.5 cc onwards. An excised size of ≥0.5 cc may arise from one large or multiple smaller biopsies. Therefore, one should consider multiple biopsies with similar prudence as the caution that is taken when performing an excisional treatment in fertile women. A meta-analysis found a 2 times increased risk of preterm birth in women undergoing loop electrosurgical excision procedure (LEEP) with ≤10 to 12 mm depth [5]. This risk further increased with increasing depth of the LEEP excision. A retrospective cohort study with 556 women found an increased risk of preterm birth in both small-medium (≤6 cc) and large (>6 cc) LEEPs compared to untreated women [5,28]. Another retrospective cohort study with 321 women showed a 3-fold increased risk of preterm birth if the excised volume was >6 cc compared to ≤6 cc [29], and a Norwegian population-based cohort study found a strong association over time from >4 cc of excised volume [30]. In contrast, a Danish population-based cohort study found an association between the depth, but not the volume, of the LEEP and the gestational age at delivery [17].

### Severity of cervical disease

While we observed that in our cohort the severity of CIN did not seem to be related to the odds of preterm birth between untreated and treated women with CIN when adjusted for the size of excision, an increased odds was seen between women with ≥CIN3 compared to women without CIN (S4 Table). These increased odds were, however, not seen between women with CIN1 or CIN2 compared to women without CIN. A retrospective cohort study with 624 women also did not find an association between CIN severity and preterm birth [31]. A Finnish population-based cohort study found, beside an increased odds for preterm birth in women with carcinoma in situ or microinvasive cancer, no increased odds with severity of CIN [32]. However, they did not adjust for excision size. Additionally, the increased odds for preterm birth was also seen in treated women with non-CIN lesions. This may implicate that the severity of cervical disease may play a role in preterm birth, although only very minor.

### Maternal outcomes

Compared to the most recent meta-analysis, we found similar findings for the association with threatened preterm birth but a less strong association with pPROM (our study: treated CIN versus no CIN OR 1.90; 95% CI 1.70 to 2.12; *P* < 0.001. Our study: treated CIN versus untreated CIN OR 1.45; 95% CI 1.25 to 1.67; *P* < 0.001. Kyrgiou and colleagues: treated CIN versus no/untreated CIN RR 2.15; 95% CI 1.48 to 3.12) [5]. However, we excluded women with induction of labor with ≤36 hours of ruptured membranes. In line with this meta-analysis, we found no association between assisted vaginal deliveries or cesarean sections and treatment of CIN (besides assisted vaginal delivery in women with treated CIN versus no CIN: OR 1.18; 95% CI 1.08 to 1.16; *P* < 0.001).

### Neonatal outcomes

The above mentioned meta-analysis showed also similar results for low birth weight [5]. Only 5 studies have outcomes on low birth weight <1,000 to 2,000 grams [17,33–36]. A retrospective cohort study found an increased risk for low birth weight and small for gestational age in women treated for ≥CIN3 compared to women without CIN [37]. Our study shows that women with treated CIN have a >2 times increased odds for low birth weight (<1,000, <1,500, <2,500, and <2,500 grams) compared to women without CIN. Our results suggest that these increased odds for low birth weight may be independent of a relationship with small for gestational age, since we found no association between Hoftiezer percentile ≤2 and treatment for CIN (Table 4 and S6 Table). Only 2 studies assessed the outcome APGAR score <7 at 5 minutes and found no statistically significant relationship [15,38], while we found a 1.5 times statistically significant increased odds in women with treated CIN. A similar increased odds was found in our study for high care and NICU admission and was comparable with the literature [5]. Perinatal death was too rare in our study to give conclusive results, but the most recent meta-analysis showed a 1.5 times increased risk for treated women compared to untreated women [5].

### Strengths and limitations

One of the strengths of our study is the use of a large population-based cohort, extracted from validated national pathology and perinatal datasets with an almost complete nationwide coverage of all pathology and pregnancies. This leads to minimal reporting, recall, and selection bias. Additionally, we included women and pregnancies over a long period of time.

For preterm birth, pPROM, and all neonatal outcomes, we only assessed women who had spontaneous births or rupture of membranes. There are many reasons for premature induction of labor, and these might be caused by underlying communal factors. We also adjusted for 19 identified potential confounders for preterm birth, reducing the likelihood that the possible correlation with preterm birth is just risk factors associated with cervical dysplasia.

We made appropriate comparison groups, by using frequency matching, and compared all 3 groups with each other: (1) women with untreated CIN versus controls; (2) women with treated CIN versus controls; and (3) women with treated CIN versus women with untreated CIN.

This study is unique in investigating associations of the volume of the excised cervical tissue of both biopsies and treatments and the odds of preterm birth with adjustment for severity of cervical disease and all other potential confounders. This gave us the opportunity to further investigate possible associations of preterm birth in women with untreated CIN. We were able to identify excision volumes for which there was an associated greater risk of preterm birth.

Limitations are the retrospective nature and the lack of information about smoking, sexual behavior, and socioeconomic status. We were unable to adjust for these factors, which are associated with both preterm birth and having CIN. However, by comparing untreated women with treated women, these possible confounders can be considered to be similar across these 2 groups.

We could not further distinct the type of excisional treatment, but the Dutch guidelines recommend treatment of high-grade CIN with LEEP [39]. Also, the excised volume could be calculated for only a select group of women, as the macroscopic description was not always (in 3 dimensions) identifiable, which potentially could give selection bias. However, the adjusted ORs of spontaneous preterm birth was not statistically significantly different between the group with known and unknown excised volume, which reduces the chance of selection bias. Furthermore, it should be kept in mind that cervical specimens are measured after formalin fixation, which may have shrunk the specimens. Therefore, the effect of the excised volume on the odds of preterm birth could be overestimated.

We could not find conclusive results on the odds of extreme premature birth <24 weeks, due to incomplete registration of extreme premature nonviable births 16 to 24 weeks, which are not mandatory to report with the municipality. This information was particularly needed to link data of Perined with PALGA.

### Clinical practice and next steps for research

With the knowledge of high regression rates of CIN1 and CIN2, especially in young women, and the increased risk for obstetrical complications after treatment, most guidelines are changing toward a more conservative approach for young women since 2012 [40,41]. However, see-and-treat approach has also become more popular over the years, which could result in higher overtreatment rates [42]. Additionally, hrHPV-based screening causes higher referral rates and more women undergoing biopsy or treatment [6], resulting in more women at risk for adverse pregnancy outcomes. With our finding that excisional procedure of both biopsy and treatment from ≥0.5 cc was associated with increased odds of preterm birth, we should optimize management of CIN in women of reproductive age. Women with a potential desire for future pregnancy should be counseled properly about the harms and benefits of both biopsy and excisional treatment. Moreover, once women with this increased risk of preterm birth become pregnant, they might benefit from closer surveillance during pregnancy to improve perinatal outcome. More studies are warranted to further investigate the role of excision of cervical tissue combined with having cervical disease on the risk of preterm birth.

## Conclusions

Both women with untreated CIN and treated CIN have an increased odds of spontaneous preterm birth compared to women without a CIN diagnosis. We observed that a volume of ≥0.5 cc excised cervical tissue was associated with preterm birth, independently from the severity of the underlying cervical disease or other potential risk factors. Caution should be taken when performing excisional treatment in women of reproductive age as well as prudence in case of multiple biopsies. Women that had a cervical excisional procedure (biopsy or treatment) with in total ≥0.5 cc excised tissue should be identified and offered close surveillance during pregnancy.

## Supporting information

S1 STROBE Checklist Strengthening the Reporting of Observational Studies in Epidemiology (STROBE) guideline. (DOCX)

S1 Table Univariable logistic regression for preterm birth per variable before each childbirth. ^a^Women with induction of labor were excluded from analysis. ^b^To adjust for multiple testing, we considered a *P* value of <0.001 statistically significant. ^c^To prevent revealing data, numbers of less than 5 are grouped together, conform the rules of CBS. *Statistically significant. CBS, Statistics Netherlands; CI, confidence interval; CIN, cervical intraepithelial neoplasia; IVF: in vitro fertilization; NA, not applicable.
(DOCX)

S2 Table Logistic regression for preterm birth for women with no CIN, untreated CIN, and treated CIN before each childbirth. ^a^With adjustment for age at childbirth, year of childbirth, urbanization, ethnicity, diabetes mellitus, maternal infection, epilepsy, psychiatric diseases, history of abortion, history of preterm birth, pregnancy by IVF, nulliparous women, pre-eclampsia, gestational diabetes, placental abruption, placenta or vasa previa, congenital diseases, intrauterine growth restriction, macrosomia, stillbirth, and fetal distress. ^b^Women with induction of labor were excluded from analysis. ^c^To adjust for multiple testing, we considered a *P* value of <0.008 statistically significant. ^d^To prevent revealing data, numbers of less than 5 are grouped together, conform the rules of CBS. *Statistically significant. CBS, Statistics Netherlands; CI, confidence interval; CIN, cervical intraepithelial neoplasia; IVF, in vitro fertilization; NA, not applicable.
(DOCX)

S3 Table Logistic regression for preterm birth per volume taken from the cervix before each childbirth. ^a^With adjustment for age at childbirth, year of childbirth, urbanization, severity of cervical disease (normal, CIN1, CIN2, or ≥CIN3), ethnicity, diabetes mellitus, maternal infection, epilepsy, psychiatric diseases, history of abortion, history of preterm birth, pregnancy by IVF, nulliparous women, pre-eclampsia, gestational diabetes, placental abruption, placenta or vasa previa, congenital diseases, intrauterine growth restriction, macrosomia, stillbirth, and fetal distress. ^b^Women with induction of labor were excluded from analysis. ^c^To adjust for multiple testing, we considered a *P* value of <0.01 statistically significant. ^d^To prevent revealing data, numbers of less than 5 are grouped together, conform the rules of CBS. *Statistically significant. CBS, Statistics Netherlands; CI, confidence interval; CIN, cervical intraepithelial neoplasia; IVF, in vitro fertilization; NA, not applicable.
(DOCX)

S4 Table Logistic regression for preterm birth per grade of CIN and volume taken from the cervix before each childbirth. ^a^With adjustment for age at childbirth, year of childbirth, urbanization, severity of cervical disease, volume taken from cervix, ethnicity, diabetes mellitus, maternal infection, epilepsy, psychiatric diseases, history of abortion, history of preterm birth, pregnancy by IVF, nulliparous women, pre-eclampsia, gestational diabetes, placental abruption, placenta or vasa previa, congenital diseases, intrauterine growth restriction, macrosomia, stillbirth, and fetal distress. ^b^Women with induction of labor were excluded from analysis. ^c^To adjust for multiple testing, we considered a *P* value of <0.007 statistically significant. *Statistically significant. CI, confidence interval; CIN, cervical intraepithelial neoplasia; IVF, in vitro fertilization; NA, not applicable.
(DOCX)

S5 Table Logistic regression for maternal outcomes for women with no CIN, untreated CIN, and treated CIN before each childbirth. ^a^With adjustment for age at childbirth, year of childbirth, urbanization, ethnicity, diabetes mellitus, maternal infection, epilepsy, psychiatric diseases, history of abortion, history of preterm birth, pregnancy by IVF, nulliparous women, pre-eclampsia, gestational diabetes, placental abruption, placenta or vasa previa, congenital diseases, intrauterine growth restriction, macrosomia, stillbirth, and fetal distress. ^b^Adjustment for pregnancy by IVF excluded. ^c^Women with induction of labor with ≤36 hours of rupture of membranes were excluded from analysis. ^d^Women with a primary cesarean section and women with the position of the baby being the indication for secondary cesarean section were excluded. ^e^To adjust for multiple testing, we considered a *P* value of <0.008 statistically significant. *Statistically significant. CI, confidence interval; CIN, cervical intraepithelial neoplasia; IVF, in vitro fertilization; pPROM, preterm premature rupture of membranes.
(DOCX)

S6 Table Logistic regression for neonatal outcomes for women no CIN, untreated CIN, and treated CIN before each childbirth. ^a^With adjustment for age at childbirth, year of childbirth, urbanization, ethnicity, diabetes mellitus, maternal infection, epilepsy, psychiatric diseases, history of abortion, history of preterm birth, pregnancy by IVF, nulliparous women, pre-eclampsia, gestational diabetes, placental abruption, placenta or vasa previa, congenital diseases, intrauterine growth restriction, macrosomia, stillbirth, and fetal distress. ^b^Women with induction of labor were excluded from analysis. ^c^To adjust for multiple testing, we considered a *P* value of <0.008 statistically significant. ^d^To prevent revealing data, numbers of less than 5 are grouped together, conform the rules of CBS. *Statistically significant. APGAR, appearance, pulse, grimace, activity and respiration; CBS, Statistics Netherlands; CI, confidence interval; CIN, cervical intraepithelial neoplasia; IVF, in vitro fertilization; NICU, neonatal intensive care unit.
(DOCX)

S1 Fig Study flowchart. CIN, cervical intraepithelial neoplasia; PALGA, the nationwide network and registry of histo- and cytopathology in the Netherlands; Perined, the Dutch perinatal registry.
(TIF)

S2 Fig Spontaneous births by gestational age for women with no CIN, untreated CIN, or treated CIN before each childbirth. The distribution between all 3 groups was statistically significantly different (all *P* < 0.001; two-sample Kolmogorov–Smirnov test). CIN, cervical intraepithelial neoplasia.
(TIF)

S3 Fig Spontaneous births by gestational age per severity of cervical disease before each childbirth. The distribution between all 4 groups was statistically significantly different (Normal vs CIN1 (*P* = 0.04), normal vs CIN2 (*P* < 0.001), normal vs CIN3 (*P* < 0.001), CIN1 vs CIN3 (*P* = 0.003)), except for CIN1 vs CIN2 (*P* = 0.11) and CIN1 vs ≥CIN3 (*P* = 0.49). (Two-sample Kolmogorov–Smirnov test). CIN, cervical intraepithelial neoplasia.
(TIF)

S4 Fig Spontaneous births by gestational age per severity of cervical disease (panels A–D) and excised volume before each childbirth. The distribution between the groups was statistically significantly different for 0.10–0.49 cc vs 4.00–8.99 cc for CIN1 (P = 0.01), 0.10–0.49 cc vs ≥9 cc for ≥CIN3 (P = 0.02), and 1.00–3.99 cc vs ≥9 cc for ≥ CIN3 (P = 0.03) (two-sample Kolmogorov–Smirnov test). CIN, cervical intraepithelial neoplasia.
(TIF)

S1 Protocol The prospective analysis plan. (DOCX)

## Abbreviations

APGAR: appearance, pulse, grimace, activity, and respiration
CBS: Statistics Netherlands
CI: confidence interval
CIN: cervical intraepithelial neoplasia
hrHPV: high-risk human papillomavirus
IVF: in vitro fertilization
KNOV: the Royal Dutch Organisation of Midwives
LEEP: loop electrosurgical excision procedure
LHV: the National Association of General Practitioners
NVK: the Dutch Association of Pediatrics
NVOG: the Dutch Society of Obstetrics and Gynaecology
OR: odds ratio
PALGA: Dutch pathology registry
Perined: Dutch perinatal database
pPROM: preterm premature rupture of membranes
PVM: Privacy Verzend Module
RR: risk ratio
STROBE: Strengthening the Reporting of Observational Studies in Epidemiology
