# Supplementary material for: Cervical intraepithelial neoplasia and the risk of spontaneous preterm birth: A Dutch population-based cohort study with 45,259 pregnancy outcomes
Source: PLoS Med. 2021 Jun 4;18(6):e1003665. doi: 10.1371/journal.pmed.1003665 (PMC8213165; doi:10.1371/journal.pmed.1003665)
Supplement: S1 Table — aWomen with induction of labor were excluded from analysis. bTo adjust for multiple testing, we considered a P value of <0.001 statistically significant. cTo prevent revealing data, numbers of less than 5 are grouped together, conform the rules of CBS. *Statistically significant. CBS, Statistics Netherlands; CI, confidence interval; CIN, cervical intraepithelial neoplasia; IVF: in vitro fertilization; NA, not applicable. (DOCX) [file pmed.1003665.s002.docx]

| **S1 Table. Univariable logistic regression for preterm birth per variable before each childbirth ^a^** | | | | |
| --- | --- | --- | --- | --- |
| **Preterm birth <37 weeks** ^a^ | **Events / total (%)** | | **Odds ratio (95%CI)** | **P-value ^b^** |
|  | 1873/ 31,224 (6.0) | |  |  |
| **Age at childbirth**, continuous | NA | | 1.03 (1.01 to 1.04) | <0.001 * |
| **Year of childbirth**, continuous | NA | | 1.02 (1.00 to 1.04) | 0.10 |
| **Urbanization**  <100,000 inhabitants vs ≥100,000 inhabitants | 1131 / 17,815 (6.3) | 742 / 13,409 (5.5) | 0.86 (0.79 to 0.95) | 0.003 |
| **Severity of cervical disease** CIN1 vs normal cytology  CIN2 vs normal cytology  ≥CIN3 vs normal cytology  CIN2 vs CIN1  ≥CIN3 vs CIN1 | 176 / 2384 (7.4)  231 / 2900 (8.0) 464 / 4971 (9.3) 231 / 2900 (8.0) 464 / 4971 (9.3) | 1002 / 20,969 (4.8) 1002 / 20,969 (4.8) 1002 / 20,969 (4.8)  176 / 2384 (7.4)  176 / 2384 (7.4) | 1.59 (1.35 to 1.88)  1.73 (1.49 to 2.00)  2.05 (1.83 to 2.30)  1.09 (0.89 to 1.33)  1.29 (1.08 to 1.55) | <0.001 *  <0.001 *  <0.001 *  0.43  0.01 |
| **Volume taken from cervix**, continuous  0.10-0.49cc vs 0cc  0.50-0.99cc vs 0cc  1.0-3.99cc vs 0cc  4.0-8.99cc vs 0cc  ≥9.0cc vs 0cc | NA  26 / 531 (4.9)  37 / 379 (9.8) 170 / 2208 (7.7) 90 / 724 (12.4) 30 / 139 (21.6) | NA 1002 / 20,969 (4.8) 1002 / 20,969 (4.8) 1002 / 20,969 (4.8)  1002 / 20,969 (4.8)  1002 / 20,969 (4.8) | 1.16 (1.13 to 1.18)  1.03 (0.69 to 1.53)  2.16 (1.53 to 3.04)  1.66 (1.40 to 1.97)  2.83 (2.25 to 3.56)  5.49 (3.64 to 8.26) | <0.001 *  0.90.  <0.001 *  <0.001 *  <0.001 *  <0.001 * |
| **Ethnicity**  Hispanic vs Caucasian  Hindu vs Caucasian  Asian vs Caucasian  Other vs Caucasian  Hispanic or Hindu vs Caucasian  Asian or other vs Caucasian | 37 / 419 (8.8)  20 / 227 (8.8)  35 / 698 (5.0) 30 / 672 (4.5) 57 / 646 (8.8) 65 / 1370 (4.7) | 1751 / 29,208 (6.0) 1751 / 29,208 (6.0) 1751 / 29,208 (6.0)  1751 / 29,208 (6.0)  1751 / 29,208 (6.0)  1751 / 29,208 (6.0) | 1.52 (1.08 to 2.14)  1.52 (0.96 to 2.40)  0.83 (0.59 to 1.17)  0.73 (0.51 to 1.06)  1.52 (1.15 to 2.00)  0.78 (0.61 to 1.01) | 0.02 .  0.08 .  0.28 .  0.10.  0.003.  0.06 |
| **Diabetes Mellitus**, yes vs no | 13 / 31 (41.9) | 1860 / 31,193 (6.0) | 11.39 (5.57 to 23.28) | <0.001 * |
| **Maternal Infection**, yes vs no | <5 / 29 (<17.2) ^c^ | 1870 / 31,195 (6.0) | 1.81 (0.55 to 5.98) | 0.33 |
| **Epilepsy**, yes vs no | <5 / 45 (<11.1) ^c^ | 1872 / 31,179 (6.0) | 0.36 (0.05 to 2.58) | 0.31 |
| **Psychiatric diseases**, yes vs no | 19 / 128 (14.8) | 1854 / 31,096 (6.0) | 2.75 (1.69 to 4.49) | <0.001 * |
| **History of abortion**, yes vs no | 29 / 539 (5.4) | 1844 / 30,685 (6.0) | 0.89 (0.61 to 1.30) | 0.54 |
| **History of preterm birth**, yes vs no | 138 / 469 (29.4) | 1735 / 30,755 (5.6) | 6.97 (5.68 to 8.56) | <0.001 * |
| **Pregnancy by IVF**, yes vs no | 46 / 387 (11.9) | 1827 / 30,837 (5.9) | 2.14 (1.57 to 2.93) | <0.001 * |
| **Nulliparous women**, yes vs no | 998 / 12,488 (8.0) | 875 / 18,736 (4.7) | 1.77 (1.61 to 1.95) | <0.001 * |
| **Pre-eclampsia**, yes vs no | 54 / 855 (6.3) | 1819 / 30,369 (6.0) | 1.06 (0.80 to 1.40) | 0.69 |
| **Gestational diabetes**, yes vs no | 52 / 443 (11.7) | 1821 / 30,781 (5.9) | 2.12 (1.58 to 2.84) | <0.001 * |
| **Placental abruption**, yes vs no | 7 / 13 (53.8) | 1866 / 31,211 (6.0) | 18.35 (6.16 to 54.65) | <0.001 * |
| **Placenta or vasa previa**, yes vs no | 7 / 22 (31.8) | 1866 / 31,202 (6.0) | 7.34 (2.99 to 18.02) | <0.001 * |
| **Congenital diseases**, yes vs no | 117 / 674 (17.4) | 1756 / 30,550 (5.7) | 3.44 (2.81 to 4.23) | <0.001 * |
| **Intrauterine growth restriction**, yes vs no | 180 / 2836 (6.3) | 1693 / 28,388 (6.0) | 1.07 (0.91 to 1.25) | 0.41 |
| **Macrosomia**, yes vs no | 20 / 301 (6.6) | 1853 / 30,923 (6.0) | 1.12 (0.71 to 1.76) | 0.64 |
| **Stillbirth**, yes vs no | 10 / 14 (71.4) | 1863 / 31,210 (6.0) | 39.38 (12.34 to 125.68) | <0.001 * |
| **Fetal distress**, yes vs no | 13 / 426 (3.1) | 1860 / 30,798 (6.0) | 0.49 (0.28 to 0.85) | 0.01 |
| ^a^ Women with induction of labor were excluded from analysis  ^b^ To adjust for multiple testing we considered a P-value of <0.001 statistically significant. ^c^ To prevent revealing data, numbers of less than 5 are grouped together, conform the rules of CBS  * Statistically significant  *Abbreviations:* CBS, Statistics Netherlands; *CI, confidence interval; CIN, cervical intraepithelial neoplasia; IVF: in vitro fertilization; NA, not applicable.* | | | | |
