# Supplementary material for: Cervical intraepithelial neoplasia and the risk of spontaneous preterm birth: A Dutch population-based cohort study with 45,259 pregnancy outcomes
Source: PLoS Med. 2021 Jun 4;18(6):e1003665. doi: 10.1371/journal.pmed.1003665 (PMC8213165; doi:10.1371/journal.pmed.1003665)
Supplement: S2 Table — aWith adjustment for age at childbirth, year of childbirth, urbanization, ethnicity, diabetes mellitus, maternal infection, epilepsy, psychiatric diseases, history of abortion, history of preterm birth, pregnancy by IVF, nulliparous women, pre-eclampsia, gestational diabetes, placental abruption, placenta or vasa previa, congenital diseases, intrauterine growth restriction, macrosomia, stillbirth, and fetal distress. bWomen with induction of labor were excluded from analysis. cTo adjust for multiple testing, we considered a P value of <0.008 statistically significant. dTo prevent revealing data, numbers of less than 5 are grouped together, conform the rules of CBS. *Statistically significant. CBS, Statistics Netherlands; CI, confidence interval; CIN, cervical intraepithelial neoplasia; IVF, in vitro fertilization; NA, not applicable. (DOCX) [file pmed.1003665.s003.docx]

| **S2 Table. Logistic regression for preterm birth for women with no CIN, untreated CIN and treated CIN before each childbirth** | | | | | | |
| --- | --- | --- | --- | --- | --- | --- |
| **Primary outcome** | **Event / total (%)** | | **Unadjusted Odds ratio (95%CI)** | **P-value ^c^** | **Adjusted ^a^ Odds ratio (95%CI)** | **P-value ^c^** |
| **Preterm birth <37 weeks** ^b^ | 1873/ 31,224 (6.0) | |  |  |  |  |
| Untreated CIN vs no CIN | 271 / 3940 (6.9) | 1002 / 20,969 (4.8) | 1.47 (1.28 to 1.69) | <0.001 * | 1.38 (1.19 to 1.60) | <0.001 * |
| Treated CIN vs no CIN | 600 / 6315 (9.5) | 1002 / 20,969 (4.8) | 2.09 (1.88 to 2.33) | <0.001 * | 2.07 (1.85 to 2.33) | <0.001 * |
| Treated CIN vs untreated CIN | 600 / 6315 (9.5) | 271 / 3940 (6.9) | 1.42 (1.22 to 1.65) | <0.001 * | 1.51 (1.29 to 1.76) | <0.001 * |
| Treated CIN ≥2x vs no CIN | 50 / 321 (15.6) | 1002 / 20,969 (4.8) | 3.68 (2.70 to 5.00) | <0.001 * | 3.66 (2.66 to 5.05) | <0.001 * |
| Treated CIN ≥2x vs untreated CIN | 50 / 321 (15.6) | 271 / 3940 (6.9) | 2.50 (1.80 to 3.46) | <0.001 * | 2.66 (1.90 to 3.72) | <0.001 * |
| Treated CIN ≥2x vs treated CIN 1x | 50 / 321 (15.6) | 550 / 5994 (9.2) | 1.83 (1.33 to 2.50) | <0.001 * | 1.83 (1.33 to 2.53) | <0.001 * |
| **Preterm birth <32 weeks** ^b^ | 228 / 31,224 (0.7) | |  | | | |
| Untreated CIN vs no CIN | 32 / 3940 (0.8) | 116 / 20,969 (0.6) | 1.47 (0.99 to 2.18) | 0.05 | 1.34 (0.88 to 2.05) | 0.18 |
| Treated CIN vs no CIN | 80 / 6315 (1.3) | 116 / 20,969 (0.6) | 2.31 (1.73 to 3.07) | <0.001 * | 2.30 (1.68 to 3.16) | <0.001 * |
| Treated CIN vs untreated CIN | 80 / 6315 (1.3) | 32 / 3940 (0.8) | 1.57 (1.04 to 2.37) | 0.03 | 1.72 (1.12 to 2.65) | 0.01 |
| Treated CIN ≥2x vs no CIN | 9 / 321 (2.8) | 116 / 20,969 (0.6) | 5.19 (2.61 to 10.31) | <0.001 * | 5.32 (2.58 to 10.95) | <0.001 * |
| Treated CIN ≥2x vs untreated CIN | 9 / 321 (2.8) | 32 / 3940 (0.8) | 3.52 (1.67 to 7.45) | 0.001 * | 3.95 (1.82 to 8.61) | <0.001 * |
| Treated CIN ≥2x vs treated CIN 1x | 9 / 321 (2.8) | 71 / 5994 (1.2) | 2.41 (1.19 to 4.86) | 0.01 | 2.47 (1.19 to 5.12) | 0.02 |
| **Preterm birth <28 weeks** ^b^ | 85 / 31,224 (0.3) | |  | | | |
| Untreated CIN vs no CIN | 12 / 3940 (0.3) | 41 / 20,969 (0.2) | 1.56 (0.82 to 2.97) | 0.17 | 1.22 (0.58 to 2.57) | 0.60 |
| Treated CIN vs no CIN | 32 / 6315 (0.5) | 41 / 20,969 (0.2) | 2.60 (1.64 to 4.13) | <0.001 * | 2.55 (1.50 to 4.35) | 0.001 * |
| Treated CIN vs untreated CIN | 32 / 6315 (0.5) | 12 / 3940 (0.3) | 1.67 (0.86 to 3.24) | 0.13 | 2.09 (0.99 to 4.40) | 0.05 |
| Treated CIN ≥2x vs no CIN | <5 / 321 (<1.6) ^d^ | 41 / 20,969 (0.2) | 6.44 (2.29 to 18.09) | <0.001 * | 7.02 (2.35 to 21.02) | <0.001 * |
| Treated CIN ≥2x vs untreated CIN | <5 / 321 (<1.6) ^d^ | 12 / 3940 (0.3) | 4.13 (1.32 to 12.88) | 0.02 | 5.72 (1.71 to 19.16) | 0.005 * |
| Treated CIN ≥2x vs treated CIN 1x | <5 / 321 (<1.6) ^d^ | 28 / 5994 (0.5) | 2.69 (0.94 to 7.71) | 0.07 | 3.03 (1.01 to 9.09) | 0.05 |
| ^a^ With adjustment for age at childbirth, year of childbirth, urbanization, ethnicity, diabetes mellitus, maternal infection, epilepsy, psychiatric diseases, history of abortion, history of preterm birth, pregnancy by IVF, nulliparous women, pre-eclampsia, gestational diabetes, placental abruption, placenta or vasa previa, congenital diseases, intrauterine growth restriction, macrosomia, stillbirth and fetal distress.  ^b^ Women with induction of labor were excluded from analysis  ^c^ To adjust for multiple testing we considered a P-value of <0.008 statistically significant  ^d^ To prevent revealing data, numbers of less than 5 are grouped together, conform the rules of CBS  * Statistically significant  *Abbreviations: CBS, Statistics Netherlands; CI, confidence interval; CIN, cervical intraepithelial neoplasia; IVF, in vitro fertilization; NA, not applicable* | | | | | | |
