# Supplementary material for: Cervical intraepithelial neoplasia and the risk of spontaneous preterm birth: A Dutch population-based cohort study with 45,259 pregnancy outcomes
Source: PLoS Med. 2021 Jun 4;18(6):e1003665. doi: 10.1371/journal.pmed.1003665 (PMC8213165; doi:10.1371/journal.pmed.1003665)
Supplement: S3 Table — aWith adjustment for age at childbirth, year of childbirth, urbanization, severity of cervical disease (normal, CIN1, CIN2, or ≥CIN3), ethnicity, diabetes mellitus, maternal infection, epilepsy, psychiatric diseases, history of abortion, history of preterm birth, pregnancy by IVF, nulliparous women, pre-eclampsia, gestational diabetes, placental abruption, placenta or vasa previa, congenital diseases, intrauterine growth restriction, macrosomia, stillbirth, and fetal distress. bWomen with induction of labor were excluded from analysis. cTo adjust for multiple testing, we considered a P value of <0.01 statistically significant. dTo prevent revealing data, numbers of less than 5 are grouped together, conform the rules of CBS. *Statistically significant. CBS, Statistics Netherlands; CI, confidence interval; CIN, cervical intraepithelial neoplasia; IVF, in vitro fertilization; NA, not applicable. (DOCX) [file pmed.1003665.s004.docx]

| **S3 Table. Logistic regression for preterm birth per volume taken from the cervix before each childbirth** | | | | | | |
| --- | --- | --- | --- | --- | --- | --- |
| **Primary outcome** | **Event / total (%)** | | **Unadjusted Odds ratio (95%CI)** | **P-value ^c^** | **Adjusted ^a^ Odds ratio (95%CI)** | **P-value ^c^** |
| **Preterm birth <37 weeks** ^b^ | 1355 / 24,950 (5.4) | |  |  |  |  |
| 0.10-0.49cc vs 0cc | 26 / 531 (4.9) | 1002 / 20,969 (4.8) | 1.03 (0.69 to 1.53) | 0.90 | 1.06 (0.69 to 1.64) | 0.79 |
| 0.50-0.99cc vs 0cc | 37 / 379 (9.8) | 1002 / 20,969 (4.8) | 2.16 (1.53 to 3.04) | <0.001 * | 2.20 (1.52 to 3.20) | <0.001 * |
| 1.00-3.99cc vs 0cc | 170 / 2208 (7.7) | 1002 / 20,969 (4.8) | 1.66 (1.40 to 1.97) | <0.001 * | 1.70 (1.40 to 2.08) | <0.001 * |
| 4.00-8.99cc vs 0cc | 90 / 724 (12.4) | 1002 / 20,969 (4.8) | 2.83 (2.25 to 3.56) | <0.001 * | 3.13 (2.44 to 4.01) | <0.001 * |
| ≥9cc vs 0cc | 30 / 139 (21.6) | 1002 / 20,969 (4.8) | 5.49 (3.64 to 8.26) | <0.001 * | 5.93 (3.86 to 9.13) | <0.001 * |
| **Preterm birth <32 weeks** ^b^ | 153 / 24,950 (0.6) | |  | | | |
| 0.10-0.49cc vs 0cc | <5 / 531(<0.9) ^d^ | 116 / 20,969 (0.6) | 1.36 (0.50 to 3.71) | 0.54 | 1.45 (0.47 to 4.43) | 0.52 |
| 0.50-0.99cc vs 0cc | <5 / 379 (<1.3) ^d^ | 116 / 20,969 (0.6) | 1.43 (0.45 to 4.53) | 0.54 | 1.41 (0.41 to 4.89) | 0.59 |
| 1.00-3.99cc vs 0cc | 16 / 2208 (0.7) | 116 / 20,969 (0.6) | 1.31 (0.78 to 2.22) | 0.31 | 1.34 (0.72 to 2.49) | 0.36 |
| 4.00-8.99cc vs 0cc | 7 / 724 (1.0) | 116 / 20,969 (0.6) | 1.76 (0.82 to 3.78) | 0.15 | 1.80 (0.76 to 4.25) | 0.18 |
| ≥9cc vs 0cc | 7 / 139 (5.0) | 116 / 20,969 (0.6) | 9.53 (4.36 to 20.83) | <0.001 * | 9.33 (3.84 to 22.68) | <0.001 * |
| **Preterm birth <28 weeks** ^b^ | 57 / 24,950 (0.2) | |  | | | |
| 0.10-0.49cc vs 0cc | <5 / 531 (<0.9) ^d^ | 41 / 20,969 (0.2) | 1.93 (0.47 to 8.00) | 0.37 | 1.70 (0.32 to 9.10) | 0.54 |
| 0.50-0.99cc vs 0cc | <5 / 379 (<1.3) ^d^ | 41 / 20,969 (0.2) | 4.07 (1.26 to 13.21) | 0.02 | 3.92 (0.95 to 16.23) | 0.06 |
| 1.00-3.99cc vs 0cc | 5 / 2208 (0.2) | 41 / 20,969 (0.2) | 1.16 (0.46 to 2.94) | 0.76 | 1.20 (0.40 to 3.62) | 0.75 |
| 4.00-8.99cc vs 0cc | <5 / 724 (<0.7) ^d^ | 41 / 20,969 (0.2) | 2.12 (0.66 to 6.88) | 0.21 | 1.83 (0.41 to 8.22) | 0.43 |
| ≥9cc vs 0cc | <5 /139 (<3.6) ^d^ | 41 / 20,969 (0.2) | 11.26 (3.45 to 36.80) | <0.001 * | 8.32 (1.82 to 38.10) | 0.006 * |
| ^a^  With adjustment for age at childbirth, year of childbirth, urbanization, severity of cervical disease (normal, CIN1, CIN2 or ≥CIN3), ethnicity, diabetes mellitus, maternal infection, epilepsy, psychiatric diseases, history of abortion, history of preterm birth, pregnancy by IVF, nulliparous women, pre-eclampsia, gestational diabetes, placental abruption, placenta or vasa previa, congenital diseases, intrauterine growth restriction, macrosomia, stillbirth and fetal distress  ^b^ Women with induction of labor were excluded from analysis  ^c^ To adjust for multiple testing we considered a P-value of <0.01 statistically significant  ^d^ To prevent revealing data, numbers of less than 5 are grouped together, conform the rules of CBS  * Statistically significant  *Abbreviations: CBS, Statistics Netherlands; CI, confidence interval; CIN, cervical intraepithelial neoplasia; IVF, in vitro fertilization; NA, not applicable* | | | | | | |
