# Supplementary material for: Cervical intraepithelial neoplasia and the risk of spontaneous preterm birth: A Dutch population-based cohort study with 45,259 pregnancy outcomes
Source: PLoS Med. 2021 Jun 4;18(6):e1003665. doi: 10.1371/journal.pmed.1003665 (PMC8213165; doi:10.1371/journal.pmed.1003665)
Supplement: S4 Table — aWith adjustment for age at childbirth, year of childbirth, urbanization, severity of cervical disease, volume taken from cervix, ethnicity, diabetes mellitus, maternal infection, epilepsy, psychiatric diseases, history of abortion, history of preterm birth, pregnancy by IVF, nulliparous women, pre-eclampsia, gestational diabetes, placental abruption, placenta or vasa previa, congenital diseases, intrauterine growth restriction, macrosomia, stillbirth, and fetal distress. bWomen with induction of labor were excluded from analysis. cTo adjust for multiple testing, we considered a P value of <0.007 statistically significant. *Statistically significant. CI, confidence interval; CIN, cervical intraepithelial neoplasia; IVF, in vitro fertilization; NA, not applicable. (DOCX) [file pmed.1003665.s005.docx]

| **S4 Table A. Logistic regression for preterm birth per grade of CIN and volume taken from the cervix before each childbirth** | | | | | | |
| --- | --- | --- | --- | --- | --- | --- |
| **Preterm birth <37 weeks** ^b^ | **Events / total (%)** | | **Unadjusted Odds ratio (95%CI)** | **P-value ^c^** | **Adjusted ^a^ Odds ratio (95%CI)** | **P-value ^c^** |
|  | 1355 / 24,950 (5.4) | |  |  |  |  |
| **Analysis including control group (i.e. including normal cytology and no volume taken from cervix)** | | | | | | |
| **Volume taken from cervix**, continuous | NA | | 1.16 (1.13 to 1.18) | <0.001 * | 1.12 (1.08 to 1.15) | <0.001 * |
| **Severity of cervical disease** | | | | | | |
| CIN1 vs normal cytology | 28 / 372 (7.5) | 1002 / 20,969 (4.8) | 1.62 (1.10 to 2.40) | 0.02 | 1.13 (0.75 to 1.71) | 0.57 |
| CIN2 vs normal cytology | 97 / 1170 (8.3) | 1002 / 20,969 (4.8) | 1.80 (1.45 to 2.24) | <0.001 * | 1.30 (1.02 to 1.67) | 0.04 |
| ≥CIN3 vs normal cytology | 228 / 2439 (9.3) | 1002 / 20,969 (4.8) | 2.06 (1.77 to 2.39) | <0.001 * | 1.44 (1.17 to 1.77) | <0.001 * |
| **Analysis excluding control group (i.e. CIN only and always volume taken from cervix)** | | | | | | |
| **Volume taken from cervix**, continuous | NA | | 1.11 (1.08 to 1.15) | <0.001 * | 1.11 (1.08 to 1.15) | <0.001 * |
| **Severity of cervical disease** | | | | | | |
| CIN2 vs CIN1 | 97 / 1170 (8.3) | 28 / 372 (7.5) | 1.11 (0.72 to 1.72) | 0.64 | 1.11 (0.70 to 1.74) | 0.66 |
| ≥CIN3 vs CIN1 | 228 / 2439 (9.3) | 28 / 372 (7.5) | 1.27 (0.84 to 1.91) | 0.26 | 1.21 (0.79 to 1.85) | 0.39 |
| ^a^ With adjustment for age at childbirth, year of childbirth, urbanization, severity of cervical disease, volume taken from cervix, ethnicity, diabetes mellitus, maternal infection, epilepsy, psychiatric diseases, history of abortion, history of preterm birth, pregnancy by IVF, nulliparous women, pre-eclampsia, gestational diabetes, placental abruption, placenta or vasa previa, congenital diseases, intrauterine growth restriction, macrosomia, stillbirth and fetal distress  ^b^ Women with induction of labor were excluded from analysis  ^c^ To adjust for multiple testing we considered a P-value of <0.007 statistically significant  * Statistically significant  *Abbreviations: CI, confidence interval; CIN, cervical intraepithelial neoplasia; IVF, in vitro fertilization; NA, not applicable* | | | | | | |

| **S4 Table B. Logistic regression for preterm birth per grade of CIN and volume taken from the cervix before each childbirth** | | | | | | |
| --- | --- | --- | --- | --- | --- | --- |
| **Preterm birth <32 weeks** ^b^ | **Events / total (%)** | | **Unadjusted Odds ratio (95%CI)** | **P-value ^c^** | **Adjusted ^a^ Odds ratio (95%CI)** | **P-value ^c^** |
|  | 153 / 24,950 (0.6) | |  |  |  |  |
| **Analysis including control group (i.e. including normal cytology and no volume taken from cervix)** | | | | | | |
| **Volume taken from cervix**, continuous | NA | | 1.16 (1.10 to 1.22) | <0.001 * | 1.17 (1.09 to 1.25) | <0.001 * |
| **Severity of cervical disease** | | | | | | |
| CIN1 vs normal cytology | 5 / 372 (1.3) | 116 / 20,969 (0.6) | 2.45 (0.99 to 6.03) | 0.05 | 1.32 (0.47 to 3.71) | 0.61 |
| CIN2 vs normal cytology | 10 / 1170 (0.9) | 116 / 20,969 (0.6) | 1.55 (0.81 to 2.97) | 0.19 | 1.10 (0.54 to 2.25) | 0.80 |
| ≥CIN3 vs normal cytology | 22 / 2439 (0.9) | 116 / 20,969 (0.6) | 1.64 (1.04 to 2.59) | 0.04 | 0.97 (0.53 to 1.79) | 0.92 |
| **Analysis excluding control group (i.e. CIN only and always volume taken from cervix)** | | | | | | |
| **Volume taken from cervix**, continuous | NA | | 1.16 (1.09 to 1.23) | <0.001 * | 1.16 (1.08 to 1.25) | <0.001 * |
| **Severity of cervical disease** | | | | | | |
| CIN2 vs CIN1 | 10 / 1170 (0.9) | 5 / 372 (1.3) | 0.63 (0.22 to 1.86) | 0.41 | 0.91 (0.28 to 3.03) | 0.88 |
| ≥CIN3 vs CIN1 | 22 / 2439 (0.9) | 5 / 372 (1.3) | 0.67 (0.25 to 1.78) | 0.42 | 0.88 (0.29 to 2.71) | 0.83 |
| ^a^ With adjustment for age at childbirth, year of childbirth, urbanization, severity of cervical disease, volume taken from cervix, ethnicity, diabetes mellitus, maternal infection, epilepsy, psychiatric diseases, history of abortion, history of preterm birth, pregnancy by IVF, nulliparous women, pre-eclampsia, gestational diabetes, placental abruption, placenta or vasa previa, congenital diseases, intrauterine growth restriction, macrosomia, stillbirth and fetal distress  ^b^ Women with induction of labor were excluded from analysis  ^c^ To adjust for multiple testing we considered a P-value of <0.007 statistically significant  * Statistically significant  *Abbreviations: CI, confidence interval; CIN, cervical intraepithelial neoplasia; IVF, in vitro fertilization; NA, not applicable* | | | | | | |

| **S4 Table C. Logistic regression for preterm birth per grade of CIN and volume taken from the cervix before each childbirth** | | | | | | |
| --- | --- | --- | --- | --- | --- | --- |
| **Preterm birth <28 weeks** ^b^ | **Events / total (%)** | | **Unadjusted Odds ratio (95%CI)** | **P-value ^c^** | **Adjusted ^a^ Odds ratio (95%CI)** | **P-value ^c^** |
|  | 57 / 24,950 (0.2) | |  |  |  |  |
| **Analysis including control group (i.e. including normal cytology and no volume taken from cervix)** | | | | | | |
| **Volume taken from cervix**, continuous | NA | | 1.18 (1.10 to 1.26) | <0.001 * | 1.18 (1.06 to 1.31) | 0.003 * |
| **Severity of cervical disease** | | | | | | |
| CIN1 vs normal cytology | <5 / 372 (<1.3) | 41 / 20,969 (0.2) | 5.55 (1.98 to 15.57) | 0.001 * | 3.31 (0.93 to 11.74) | 0.06 |
| CIN2 vs normal cytology | <5/ 1170 (<0.4) | 41 / 20,969 (0.2) | 1.75 (0.63 to 4.90) | 0.29 | 1.32 (0.42 to 4.13) | 0.63 |
| ≥CIN3 vs normal cytology | 8 / 2439 (0.3) | 41 / 20,969 (0.2) | 1.68 (0.79 to 3.59) | 0.18 | 0.97 (0.34 to 2.78) | 0.95 |
| **Analysis excluding control group (i.e. CIN only and always volume taken from cervix)** | | | | | | |
| **Volume taken from cervix**, continuous | NA | | 1.16 (1.06 to 1.27) | <0.001 * | 1.16 (1.04 to 1.29) | 0.01 |
| **Severity of cervical disease** | | | | | | |
| CIN2 vs CIN1 | <5 / 1170 (<0.4) | <5 / 372 (<1.3) | 0.32 (0.08 to 1.27) | 0.10 | 0.42 (0.09 to 1.96) | 0.27 |
| ≥CIN3 vs CIN1 | 8 / 2439 (0.3) | <5 / 372 (<1.3) | 0.30 (0.09 to 1.01) | 0.05 | 0.37 (0.09 to 1.52) | 0.17 |
| ^a^ With adjustment for age at childbirth, year of childbirth, urbanization, severity of cervical disease, volume taken from cervix, ethnicity, diabetes mellitus, maternal infection, epilepsy, psychiatric diseases, history of abortion, history of preterm birth, pregnancy by IVF, nulliparous women, pre-eclampsia, gestational diabetes, placental abruption, placenta or vasa previa, congenital diseases, intrauterine growth restriction, macrosomia, stillbirth and fetal distress  ^b^ Women with induction of labor were excluded from analysis  ^c^ To adjust for multiple testing we considered a P-value of <0.007 statistically significant  * Statistically significant  *Abbreviations: CI, confidence interval; CIN, cervical intraepithelial neoplasia; IVF, in vitro fertilization; NA, not applicable* | | | | | | |
