# Supplementary material for: Cervical intraepithelial neoplasia and the risk of spontaneous preterm birth: A Dutch population-based cohort study with 45,259 pregnancy outcomes
Source: PLoS Med. 2021 Jun 4;18(6):e1003665. doi: 10.1371/journal.pmed.1003665 (PMC8213165; doi:10.1371/journal.pmed.1003665)
Supplement: S5 Table — aWith adjustment for age at childbirth, year of childbirth, urbanization, ethnicity, diabetes mellitus, maternal infection, epilepsy, psychiatric diseases, history of abortion, history of preterm birth, pregnancy by IVF, nulliparous women, pre-eclampsia, gestational diabetes, placental abruption, placenta or vasa previa, congenital diseases, intrauterine growth restriction, macrosomia, stillbirth, and fetal distress. bAdjustment for pregnancy by IVF excluded. cWomen with induction of labor with ≤36 hours of rupture of membranes were excluded from analysis. dWomen with a primary cesarean section and women with the position of the baby being the indication for secondary cesarean section were excluded. eTo adjust for multiple testing, we considered a P value of <0.008 statistically significant. *Statistically significant. CI, confidence interval; CIN, cervical intraepithelial neoplasia; IVF, in vitro fertilization; pPROM, preterm premature rupture of membranes. (DOCX) [file pmed.1003665.s006.docx]

| **S5 Table. Logistic regression for maternal outcomes for women with no CIN, untreated CIN and treated CIN before each childbirth** | | | | | | |
| --- | --- | --- | --- | --- | --- | --- |
| **Maternal outcomes** | **Event / total (%)** | | **Unadjusted Odds ratio (95%CI)** | **P-value ^e^** | **Adjusted ^a^ Odds ratio (95%CI)** | **P-value ^e^** |
| **Spontaneous conception** ^b^ | 27,938 / 30,087 (92.9) | |  |  |  |  |
| Untreated CIN vs no CIN | 3710 / 4017 (92.4) | 18,336 / 19,668 (93.2) | 0.88 (0.77 to 1.00) | 0.88 | 1.27 (1.11 to 1.45) | 0.001 * |
| Treated CIN vs no CIN | 5892 / 6402 (92.0) | 18,336 / 19,668 (93.2) | 0.84 (0.76 to 0.93) | 0.84 | 1.19 (1.07 to 1.33) | 0.002 * |
| Treated CIN vs untreated CIN | 5892 / 6402 (92.0) | 3710 / 4017 (92.4) | 0.96 (0.83 to 1.12) | 0.55 | 0.94 (0.81 to 1.09) | 0.43 |
| Treated CIN ≥2x vs no CIN | 332 / 369 (90.0) | 18,336 / 19,668 (93.2) | 0.65 (0.46 to 0.92) | 0.02 | 0.98 (0.69 to 1.40) | 0.92 |
| Treated CIN ≥2x vs untreated CIN | 332 / 369 (90.0) | 3710 / 4017 (92.4) | 0.74 (0.52 to 1.06) | 0.10 | 0.78 (0.54 to 1.12) | 0.18 |
| Treated CIN ≥2x vs treated CIN 1x | 332 / 369 (90.0) | 5560 / 6033 (92.2) | 0.76 (0.54 to 1.09) | 0.13 | 0.81 (0.57 to 1.17) | 0.26 |
| **Threatened preterm birth** | 1247 / 45,259 (2.8) | |  | | | |
| Untreated CIN vs no CIN | 192 / 5940 (3.2) | 659 / 29,907 (2.2) | 1.48 (1.26 to 1.75) | <0.001 * | 1.53 (1.29 to 1.81) | <0.001 * |
| Treated CIN vs no CIN | 396 / 9412 (4.2) | 659 / 29,907 (2.2) | 1.95 (1.72 to 2.21) | <0.001 * | 2.08 (1.81 to 2.38) | <0.001 * |
| Treated CIN vs untreated CIN | 396 / 9412 (4.2) | 192 / 5940 (3.2) | 1.32 (1.10 to 1.57) | 0.002 * | 1.36 (1.14 to 1.62) | 0.001 * |
| Treated CIN ≥2x vs no CIN | 31 / 505 (6.1) | 659 / 29,907 (2.2) | 2.90 (2.00 to 4.21) | <0.001 * | 3.12 (2.14 to 4.56) | <0.001 * |
| Treated CIN ≥2x vs untreated CIN | 31 / 505 (6.1) | 192 / 5940 (3.2) | 1.96 (1.33 to 2.89) | 0.001 * | 2.04 (1.38 to 3.03) | <0.001 * |
| Treated CIN ≥2x vs treated CIN 1x | 31 / 505 (6.1) | 365 / 8907 (4.1) | 1.53 (1.05 to 2.23) | 0.03 | 1.55 (1.06 to 2.26) | 0.03 |
| **pPROM** ^c^ | 1971 / 30,740 (6.4) | |  | | | |
| Untreated CIN vs no CIN | 292 / 3953 (7.4) | 1045 / 20,386 (5.1) | 1.48 (1.29 to 1.69) | <0.001 * | 1.31 (1.13 to 1.51) | <0.001 * |
| Treated CIN vs no CIN | 634 / 6401 (9.9) | 1045 / 20,386 (5.1) | 2.04 (1.84 to 2.26) | <0.001 * | 1.90 (1.70 to 2.12) | <0.001 * |
| Treated CIN vs untreated CIN | 634 / 6401 (9.9) | 292 / 3953 (7.4) | 1.38 (1.19 to 1.59) | <0.001 * | 1.45 (1.25 to 1.67) | <0.001 * |
| Treated CIN ≥2x vs no CIN | 54 / 337 (16.0) | 1045 / 20,386 (5.1) | 3.53 (2.62 to 4.76) | <0.001 * | 3.30 (2.43 to 4.48) | <0.001 * |
| Treated CIN ≥2x vs untreated CIN | 54 / 337 (16.0) | 292 / 3953 (7.4) | 2.39 (1.75 to 3.28) | <0.001 * | 2.52 (1.83 to 3.48) | <0.001 * |
| Treated CIN ≥2x vs treated CIN 1x | 54 / 337 (16.0) | 580 / 6064 (9.6) | 1.80 (1.33 to 2.44) | <0.001 * | 1.80 (1.32 to 2.46) | <0.001 * |
| **Assisted vaginal delivery** | 3941 / 44,556 (8.8) | |  | | | |
| Untreated CIN vs no CIN | 556 / 5832 (9.5) | 2453 / 29,482 (8.3) | 1.16 (1.05 to 1.28) | 0.002 * | 1.04 (0.94 to 1.16) | 0.42 |
| Treated CIN vs no CIN | 932 / 9242 (10.1) | 2453 / 29,482 (8.3) | 1.24 (1.14 to 1.34) | <0.001 * | 1.18 (1.08 to 1.29) | <0.001 * |
| Treated CIN vs untreated CIN | 932 / 9242 (10.1) | 556 / 5832 (9.5) | 1.06 (0.95 to 1.19) | 0.27 | 1.13 (1.01 to 1.27) | 0.04 |
| Treated CIN ≥2x vs no CIN | 42 / 498 (8.4) | 2453 / 29,482 (8.3) | 1.02 (0.74 to 1.40) | 0.93 | 0.95 (0.68 to 1.32) | 0.75 |
| Treated CIN ≥2x vs untreated CIN | 42 / 498 (8.4) | 556 / 5832 (9.5) | 0.87 (0.63 to 1.21) | 0.42 | 0.91 (0.64 to 1.28) | 0.57 |
| Treated CIN ≥2x vs treated CIN 1x | 42 / 498 (8.4) | 890 / 8744 (10.2) | 0.81 (0.59 to 1.12) | 0.21 | 0.79 (0.57 to 1.11) | 0.18 |
| **Caesarean section** ^d^ | 3640 / 44,157 (8.2) | |  | | | |
| Untreated CIN vs no CIN | 518 / 5777 (9.0) | 2343 / 29,231 (8.0) | 1.13 (1.02 to 1.25) | 0.02 | 0.94 (0.85 to 1.04) | 0.23 |
| Treated CIN vs no CIN | 779 / 9149 (8.5) | 2343 / 29,231 (8.0) | 1.07 (0.98 to 1.16) | 0.13 | 0.91 (0.83 to 0.99) | 0.03 |
| Treated CIN vs untreated CIN | 779 / 9149 (8.5) | 518 / 5777 (9.0) | 0.95 (0.84 to 1.06) | 0.34 | 0.97 (0.86 to 1.09) | 0.55 |
| Treated CIN ≥2x vs no CIN | 41 / 488 (8.4) | 2343 / 29,231 (8.0) | 1.05 (0.76 to 1.45) | 0.76 | 0.86 (0.62 to 1.19) | 0.37 |
| Treated CIN ≥2x vs untreated CIN | 41 / 488 (8.4) | 518 / 5777 (9.0) | 0.93 (0.67 to 1.30) | 0.67 | 0.92 (0.66 to 1.28) | 0.61 |
| Treated CIN ≥2x vs treated CIN 1x | 41 / 488 (8.4) | 738 / 8661 (8.5) | 0.99 (0.71 to 1.37) | 0.93 | 0.95 (0.68 to 1.32) | 0.75 |
| ^a^ With adjustment for age at childbirth, year of childbirth, urbanization, ethnicity, diabetes mellitus, maternal infection, epilepsy, psychiatric diseases, history of abortion, history of preterm birth, pregnancy by IVF, nulliparous women, pre-eclampsia, gestational diabetes, placental abruption, placenta or vasa previa, congenital diseases, intrauterine growth restriction, macrosomia, stillbirth and fetal distress  ^b^ Adjustment for pregnancy by IVF excluded  ^c^ Women with induction of labor with ≤36 hours of rupture of membranes were excluded from analysis  ^d^ Women with a primary caesarean section and women with the position of the baby being the indication for secondary caesarean section were excluded  ^e^ To adjust for multiple testing we considered a P-value of <0.008 statistically significant  * Statistically significant  *Abbreviations: CI, confidence interval; CIN, cervical intraepithelial neoplasia; IVF, in vitro fertilization; pPROM, preterm premature rupture of membranes* | | | | | | |
