# Supplementary figures and images for: Cervical intraepithelial neoplasia and the risk of spontaneous preterm birth: A Dutch population-based cohort study with 45,259 pregnancy outcomes
Source: PLoS Med. 2021 Jun 4;18(6):e1003665. doi: 10.1371/journal.pmed.1003665 (PMC8213165; doi:10.1371/journal.pmed.1003665)

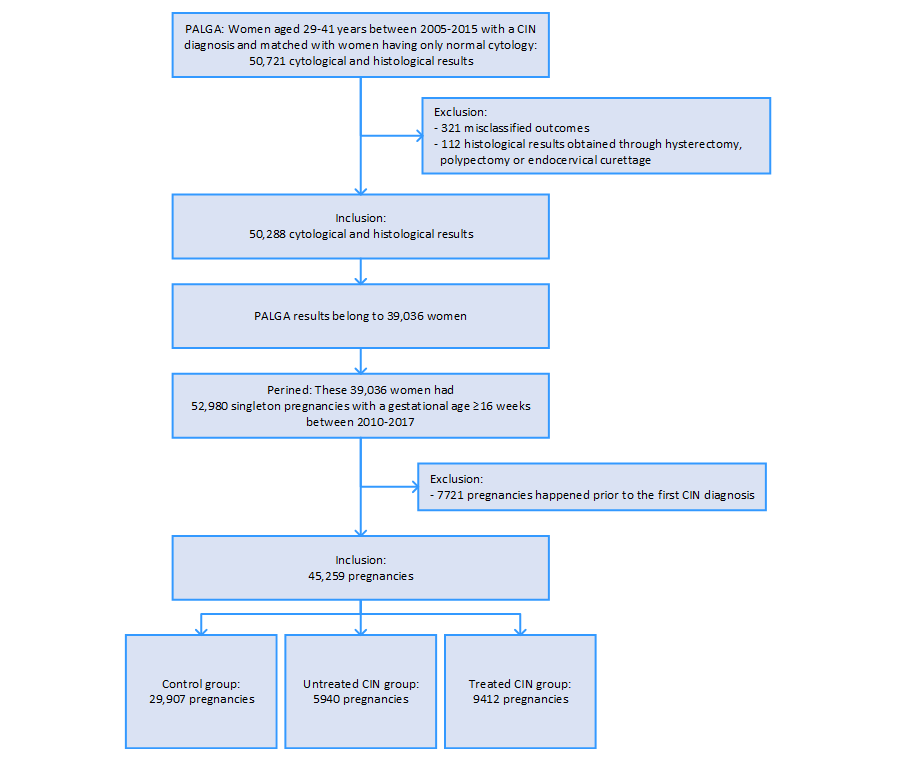

Supplement: S1 Fig — CIN, cervical intraepithelial neoplasia; PALGA, the nationwide network and registry of histo- and cytopathology in the Netherlands; Perined, the Dutch perinatal registry. (TIF) [file pmed.1003665.s008.tif]

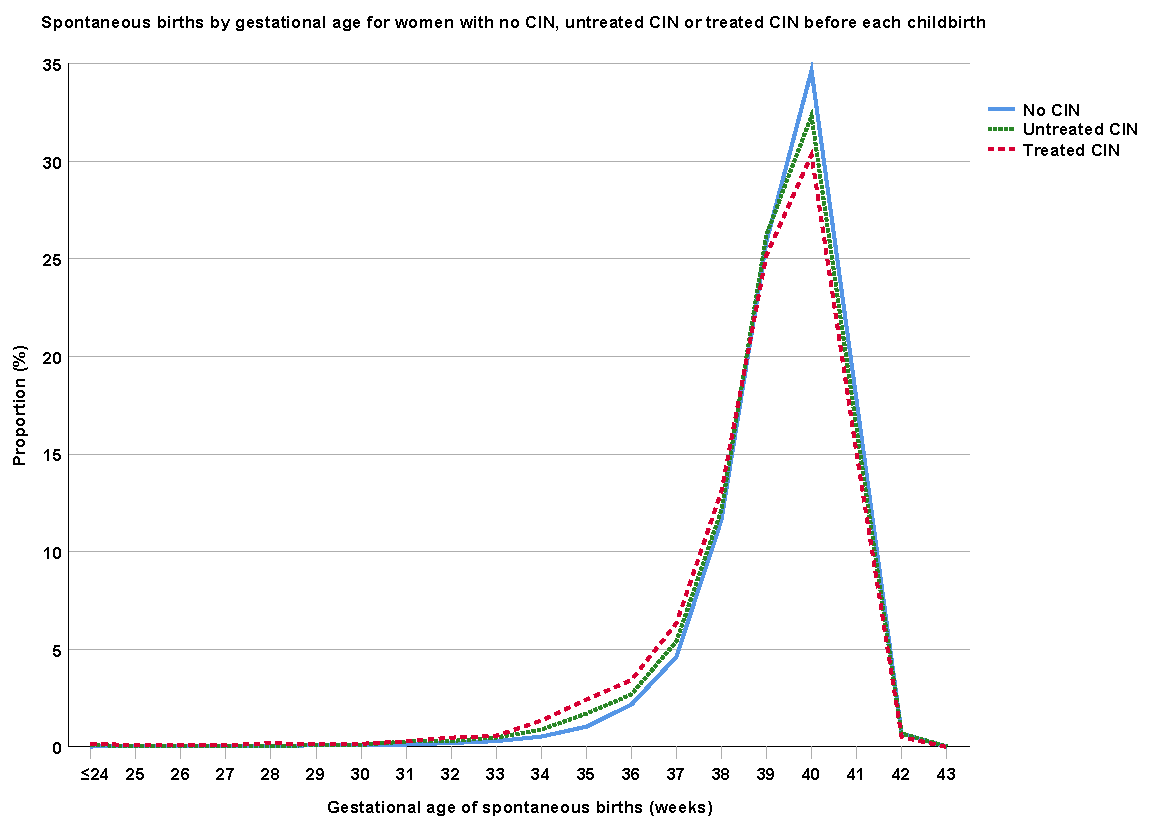

Supplement: S2 Fig — The distribution between all 3 groups was statistically significantly different (all P < 0.001; two-sample Kolmogorov–Smirnov test). CIN, cervical intraepithelial neoplasia. (TIF) [file pmed.1003665.s009.tif]

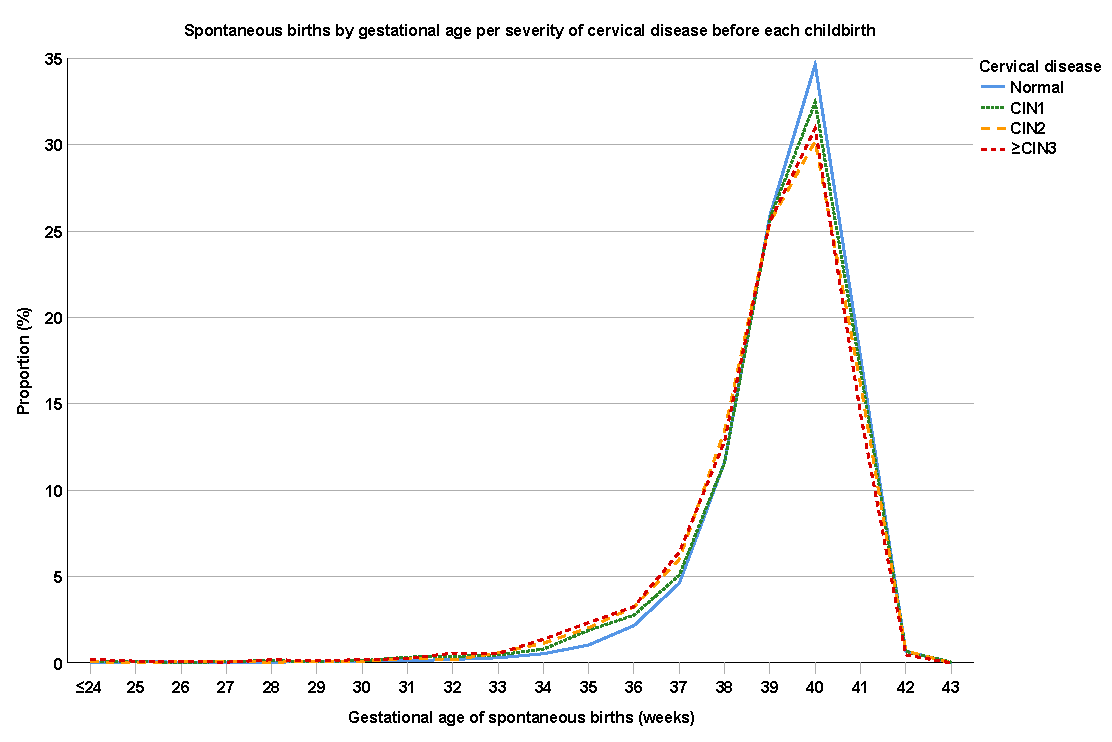

Supplement: S3 Fig — The distribution between all 4 groups was statistically significantly different (Normal vs CIN1 (P = 0.04), normal vs CIN2 (P < 0.001), normal vs CIN3 (P < 0.001), CIN1 vs CIN3 (P = 0.003)), except for CIN1 vs CIN2 (P = 0.11) and CIN1 vs ≥CIN3 (P = 0.49). (Two-sample Kolmogorov–Smirnov test). CIN, cervical intraepithelial neoplasia. (TIF) [file pmed.1003665.s010.tif]

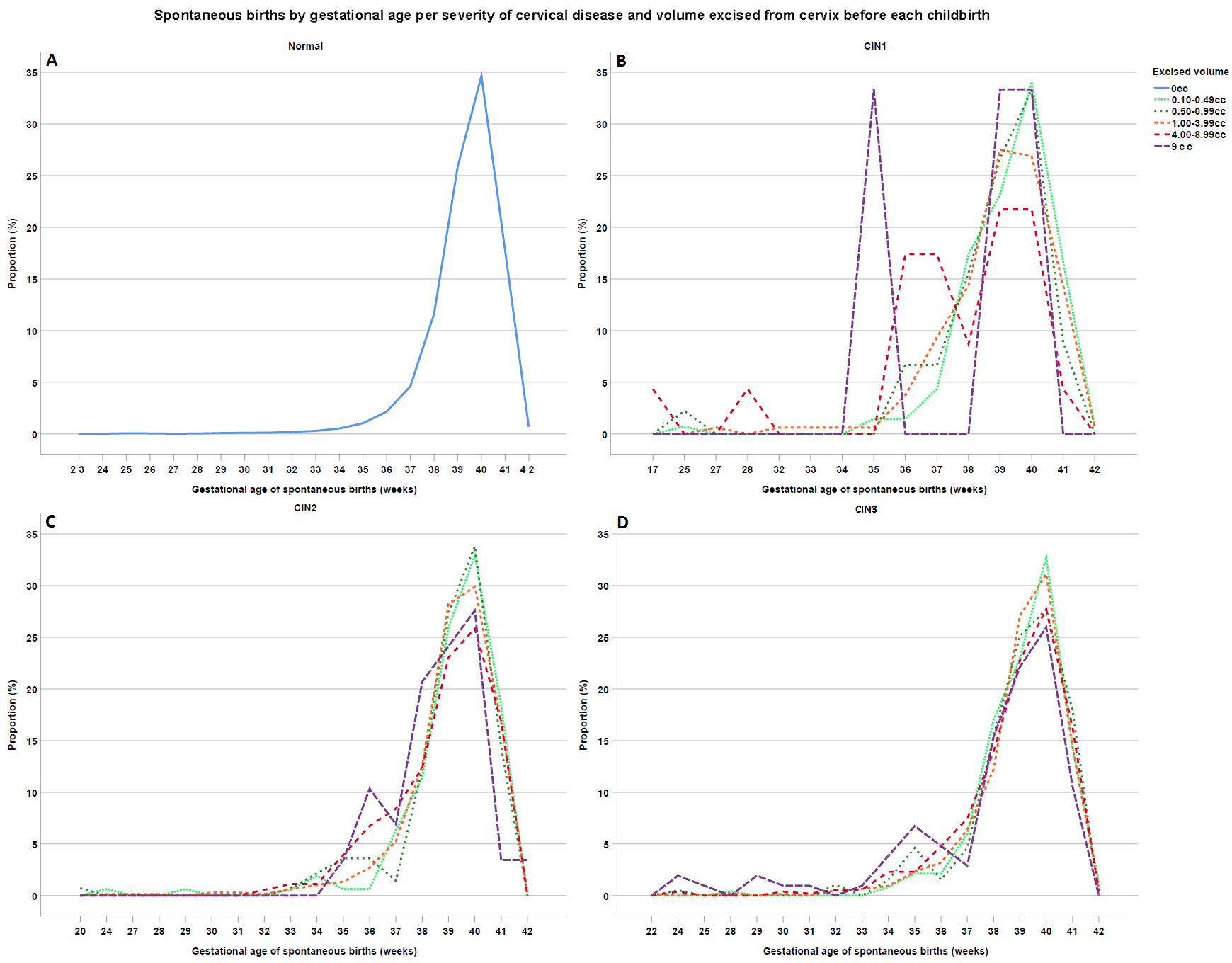

Supplement: S4 Fig — Spontaneous births by gestational age per severity of cervical disease (panels A–D) and excised volume before each childbirth. The distribution between the groups was statistically significantly different for 0.10–0.49 cc vs 4.00–8.99 cc for CIN1 (P = 0.01), 0.10–0.49 cc vs ≥9 cc for ≥CIN3 (P = 0.02), and 1.00–3.99 cc vs ≥9 cc for ≥ CIN3 (P = 0.03) (two-sample Kolmogorov–Smirnov test). CIN, cervical intraepithelial neoplasia. (TIF) [file pmed.1003665.s011.tif]
