# Supplementary material for: Cervical intraepithelial neoplasia and the risk of spontaneous preterm birth: A Dutch population-based cohort study with 45,259 pregnancy outcomes
Source: PLoS Med. 2021 Jun 4;18(6):e1003665. doi: 10.1371/journal.pmed.1003665 (PMC8213165; doi:10.1371/journal.pmed.1003665)
Supplement: S1 Protocol — (DOCX) [file pmed.1003665.s012.docx]

**Supporting information: S1 Protocol

Prospective analysis plan**

**Cervical intraepithelial neoplasia and the risk of spontaneous preterm birth: a Dutch population-based cohort study with 45,259 pregnancy outcomes**
Diede L Loopik; Joris van Drongelen; Ruud LM Bekkers; Quirinus JM Voorham; Willem JG Melchers; Leon FAG Massuger; Folkert J van Kemenade; Albert G Siebers

**Project title**
(Un)treated cervical intraepithelial neoplasia (CIN) and adverse obstetrical and perinatal outcomes.

**Background**

Cervical cancer screening has been in place in the Netherlands for several decades. Aim of the screening program is to identify and treat premalignant cervical lesions before they progress to cancer. This has led to a strong reduction in cervical cancer incidence and mortality. However, the downside of this screening is unnecessary referral of many women without any abnormalities or lesions that only have very low risk of progressing to cancer. This downside is even more pronounced in the new screening program where high-risk human papillomavirus (hrHPV) testing has replaced primary cytology testing. Most high-risk cervical lesions but inevitably also many lesions with a lower risk are treated by large loop excision of the transformation zone (LLETZ). Various studies and meta-analyses point to an increased risk of perinatal complications such as preterm delivery, low birth weight, premature rupture of membranes and caesarean delivery after LLETZ treatment. Therefore, overtreatment of fertile women has main priority for policymakers. However, it is unknown whether this increased risk is only due to the excision procedure itself, to the underlying cervical intraepithelial neoplasia (CIN) or to secondary risk factors which are associated with both preterm birth and CIN. The purpose of this study is to investigate the association between untreated CIN, treated CIN and adverse obstetrical and perinatal outcomes.

**Research question**

What is the association between women with (un)treated CIN and risk of adverse obstetrical and perinatal outcomes?

**Hypothesis**
Women with a history of CIN, and especially women who have been treated for CIN, are at increased risk of premature birth. The depth of treatment and number of treatments will increase the risk of premature birth.

**Methods**

To answer the research question we started to explore the possibilities of linking treatment data of CIN from the Dutch pathology database (PALGA) with the Dutch perinatal database (Perined). For this purpose involvement of a trusted third party (ZorgTTP) and Statistics Netherlands (CBS) is considered necessary for linking PALGA data to perinatal outcome data from Perined. A mandatory condition is that personal identifiers of the mother are available and that pseudonyms of PALGA and Perined can be harmonized. Furthermore the procedure must meet all current privacy legislation issues.
A specially designed ‘Privacy Verzend Module’ (PVM) delivered by ZorgTTP will be used to convert ZorgTTP pseudonyms (based on social security number (BSN)) into CBS BSN-pseudonyms and to send them to CBS. At CBS the BSN-pseudonyms will be linked and the corresponding BSN will be assigned. Based on the secondary keys (such as date of birth, postal code and sex) the linking results will be checked. When identical the variable ‘Rinpersoon’ is added to the dataset. Based on ‘Rinpersoon’ the PALGA data are linked to the Perined data (Rinpersoon_mother).
After linkage of the PALGA and Perined data at CBS, the researchers will analyze only anonymized data within the CBS ICT environment.

**PALGA selection**Three groups will be selected in PALGA.

Group 1) Women with untreated CIN
Inclusion criteria:
 - Period: 01-01-2005 until 31-12-2015
 - Age: 29-41 years at date of CIN diagnosis (so women invited for three screening rounds are
 included: age 30, 35 and 40)
 - Histologic diagnosis: CIN1, CIN2, CIN3 or AIS diagnosed via biopsy
 Exclusion criteria: histology through treatment, such as LLETZ, ablation, hysterectomy or other.
Group 2) Women with treated CIN
Inclusion criteria:
 - Period: 01-01-2005 until 31-12-2015
 - Age: 29-41 years at date of CIN diagnosis (so women invited for three screening rounds are
 included: age 30, 35 and 40)
 - Histologic diagnosis: CIN1, CIN2, CIN3 or AIS diagnosed via biopsy or treatment
 - Requirement: at least one treatment outcome through LLETZ
Group 3) Control group: women without CIN
Inclusion criteria:
 - Period: 01-01-2005 until 31-12-2015
 - Cytologic diagnosis: normal cytology
Exclusion criteria:
 - Cytologic diagnosis: ASCUS or more in history or follow-up
 - Histologic diagnosis: CIN1 or more in history or follow-up
Group 3 will be matched (1:3?) with group 1 and 2 based on age at and year of diagnosis and urbanization.

Variables

- Age of women during cytology/biopsy/treatment
- Date of cytology/biopsy/treatment
- Number of biopsies/treatments
- Histologic diagnosis
- Macroscopic description to calculate the size/volume of the biopsy/treatment by a datamining
 procedure.
- Type of treatment cannot be further specified

**Perined selection**- Period: 01-01-2010 until 31-12-2018
- Pregnancies between 16-24 weeks could be in the database, but this is not required
- Pregnancies of 24 weeks and more are mandatory in the database

Maternal variables
- Conception (spontaneous or with medical help)
- General history
- Obstetrical history
- Gravidity
- Parity
- Singleton or multiple pregnancy (twins, triplets etc)
- Gestation in days and weeks
- Age of the mother at childbirth (calculation through date of childbirth and age at histology date?)
- Date of childbirth
- Start of delivery (spontaneous, iatrogenic)
- Way of delivery (spontaneous, assisted vaginal delivery, caesarean section)
- Presenting part during delivery
- Problems during current pregnancy

Neonatal variables
- Birth weight in grams
- Apgar score after 5 minutes
- Congenital diseases
- Admission after birth (high care, intensive care, post-ICHC; period of admission in days)
- Perinatal death (gestation, antepartum/durante partu/postpartum)

Risk factors for preterm birth
- Ethnicity
- Smoking
- Immunosuppressive medication
- BMI
- General history: infectious diseases, auto-immune diseases, organ-transplantation, diabetes
 mellitus, epilepsy, psychiatric diseases, thyroid disorders
- Obstetrical history: curettage in history, history of abortion, history of preterm birth
- Short interval between pregnancies
- Conception through IVF/ICSI/egg donation
- Multiple pregnancy (twins, triplets etc)
- Nulliparous women
- Problems during current pregnancy: maternal infection, pre-eclampsia, gestational diabetes,
 placental problems
- Congenital diseases

Date of histology and date of childbirth will be used to make a selection of women who were treated before and after their pregnancy to additionally compare perinatal outcomes before treatment and after treatment.

**Analyses**
Primary outcome
The odds ratio of premature birth (<28/<32/<37 weeks) will be calculated between the three groups
Secondary outcomes
Threatened premature birth, premature rupture of membranes, mode of delivery, low birth weight, admission to NICU, APGAR score, perinatal mortality
Sub analysis
Will be done for severity of grade, number of biopsies/treatments and size of biopsy and/or LLETZ
Confounders
There will be adjusted for potential confounders, such as gemelli pregnancy, premature birth in history, induction of labor, IVF/ICSI pregnancy, pre-eclampsia, diabetes mellitus, diabetes gravidarum, age, smoking, BMI, immunosuppressive drug use, congenital disorders, ethnicity etc.

**Parties involved**

Radboudumc, Erasmusmc, BBMRI, Perined, PALGA, ZorgTTP, CBS

**Changes in the plan:**During data-preparation
- We decided to exclude all multiple pregnancies (twins, triplets etc)
**-** We decided to include women with only treatment after the pregnancy, but biopsy-proved CIN before the pregnancy in group 1 (untreated CIN)
- We decided to only use the spontaneous births for our analysis
- We could not adjust for all potential confounders, as not all these data was (well) documented in Perined, such as smoking, BMI, immunosuppressive drug use, sexual behavior and socio-economic status
- We could only calculate the size of the excised volume for a select group of women, as the macroscopic description was not always (in three dimensions) identifiable. We could, therefore, not use this variable in the main multivariate analysis.
During data-analysis
- The two selected groups from PALGA were matched with a control group, but after linking this data with Perined and looking per pregnancy instead of per women the groups were not completely equal anymore based on these matching criteria. We therefore decided, for example, to also include ‘urbanization’ in the multivariate analysis.

**Changes after peer review:**
- We included unadjusted analyses for all outcomes in the supporting information
- We included the data of the dose response relationship between increasing severity of CIN or more aggressive treatment with preterm birth of <32 weeks and <28 weeks, instead of only <37 weeks
- Instead of only dichotomized outcomes for preterm birth, we also included a quantitative measure of prematurity in days/weeks of gestation
- We divided supplementary figure 4 into a four panel graph
